# The role of blockchain to secure internet of medical things

**DOI:** 10.1038/s41598-024-68529-x

**Published:** 2024-08-08

**Authors:** Yazeed Yasin Ghadi, Tehseen Mazhar, Tariq Shahzad, Muhammad Amir khan, Alaa Abd-Alrazaq, Arfan Ahmed, Habib Hamam

**Affiliations:** 1 Department of Computer Science and Software Engineering, Al Ain University, Abu Dhabi, 15322 UAE; 2 Department of Computer Science, Virtual University of Pakistan, Lahore, 55150 Pakistan; 3 Department of Computer Science, COMSATS University Islamabad, Sahiwal Campus, Sahiwal, 57000 Pakistan; 4 School of Computing Sciences, College of Computing, Informatics and Mathematics, Universiti Teknologi MARA, 40450 Shah Alam, Selangor Malaysia; 5 AI Center for Precision Health, Weill Cornell Medicine-Qatar, Doha, Qatar; 6 Faculty of Engineering, Université de Moncton, Moncton, NB E1A3E9 Canada; 7 School of Electrical Engineering, Department of Electrical and Electronic Engineering Science, University of Johannesburg, Johannesburg, 2006 South Africa; 8 Hodmas University College, Taleh Area, Mogadishu, Somalia; 9 Bridges for Academic Excellence, Tunis, Tunisia

**Keywords:** IoMT, Blockchain, IoT, Challenges, Integration, Solutions, Health care, Medical research, Energy science and technology, Engineering

## Abstract

This study explores integrating blockchain technology into the Internet of Medical Things (IoMT) to address security and privacy challenges. Blockchain’s transparency, confidentiality, and decentralization offer significant potential benefits in the healthcare domain. The research examines various blockchain components, layers, and protocols, highlighting their role in IoMT. It also explores IoMT applications, security challenges, and methods for integrating blockchain to enhance security. Blockchain integration can be vital in securing and managing this data while preserving patient privacy. It also opens up new possibilities in healthcare, medical research, and data management. The results provide a practical approach to handling a large amount of data from IoMT devices. This strategy makes effective use of data resource fragmentation and encryption techniques. It is essential to have well-defined standards and norms, especially in the healthcare sector, where upholding safety and protecting the confidentiality of information are critical. These results illustrate that it is essential to follow standards like HIPAA, and blockchain technology can help ensure these criteria are met. Furthermore, the study explores the potential benefits of blockchain technology for enhancing inter-system communication in the healthcare industry while maintaining patient privacy protection. The results highlight the effectiveness of blockchain’s consistency and cryptographic techniques in combining identity management and healthcare data protection, protecting patient privacy and data integrity. Blockchain is an unchangeable distributed ledger system. In short, the paper provides important insights into how blockchain technology may transform the healthcare industry by effectively addressing significant challenges and generating legal, safe, and interoperable solutions. Researchers, doctors, and graduate students are the audience for our paper.

## Introduction

### Initial application

The initial application of blockchain technology was Bitcoin in 2008. Three characteristics make blockchain different from other solutions: decentralization, transparency, and confidentiality. Its possible application in other data-centric industries, including healthcare, has drawn interest. IBM believes the healthcare sector will be seriously impacted in three areas: the decentralized interchange of electronic health information (EHRs), clinical trial administration, and regulatory compliance. Because it permits data to be transferred between devices via innovative wireless channels, the Internet of Things (IoT) is essential to the healthcare industry.

The IoMT refers to this network’s focus on patient care. Thanks to the collection of physiological data by Internet of Medical Things devices, medical experts may make precise diagnoses. Due to its numerous benefits, such as data security and simpler management of Internet of Things devices, blockchain has established itself as a dependable and decentralized platform. Blockchain technology has secure access, data storage, and medical research applications. Attention to patient privacy regulations and data-sharing protocols facilitates accurate data visualization, which is especially helpful during pandemics such as the COVID-19 outbreak. By combining blockchain technology with IoMT devices, patient privacy and data distribution are improved. Reliability in smart contracts helps to prevent data manipulation. Blockchain’s decentralized storage enhances data collection, sharing, and storage transparency The emergence of blockchain technology marked a significant milestone with the introduction of the digital currency Bitcoin in 2008^1^. Its foundational attributes of transparency, confidentiality, and decentralization set blockchain apart from other technologies. By March 19, 2019, approximately 400 million Bitcoin transactions had been executed successfully, serving as a compelling illustration of the myriad possibilities offered by blockchain technology^2^. As a result of this groundbreaking success, there has been a burgeoning interest in exploring the potential applications of blockchain technology across various data-centric industries, notably in the healthcare field^3^. A leading technology company, IBM, has advocated adopting blockchain in healthcare^4^. They anticipate that blockchain technology will substantially influence the healthcare sector, particularly in critical areas such as clinical trial management, regulatory compliance, and creating a decentralized mechanism for the secure exchange of electronic health records (EHRs)^5^.

### Recent advances in Blockchain for EMR

HealthChain, an EMR application developed as a permission, private blockchain network, leverages IBM Blockchain’s Hyperledger Fabric and is deployed on Bluemix. The modular architecture of Hyperledger Fabric enables HealthChain to ensure health data confidentiality, scalability, and security^6^. Health Chain also incorporates chain codes (smart contracts) to control authorisations and access privileges within the blockchain network^7^. Additionally, Ancile, built on the Ethereum blockchain platform, utilizes smart contracts to achieve access control, data security, privacy, and interoperability of electronic medical records^8^. MedRec and the medical data preservation system (DPS) developed by Li et al. are notable examples that utilize the Ethereum blockchain platform to implement EMR^9–11^. Other blockchain-based EMR applications include MedBlock^12^, BlockHIE^13^, FHIRChain^14^, and MeDShare^15^, further illustrating the diversity and potential of blockchain technologies in enhancing EMR systems.

By the year 2022, it is expected that the market for blockchain technology will reach its peak within the healthcare business. This prediction highlights the possible effect that blockchain technology will have on the healthcare industry^16^. This projection reflects the growing recognition of blockchain’s potential to revolutionize various facets of healthcare, from enhancing data security to streamlining administrative processes. In essence, the foundational principles of transparency, confidentiality, and decentralization that underpin blockchain technology have sparked a wave of enthusiasm and exploration across multiple industries, particularly healthcare. The potential to enhance the management of clinical trials, ensure regulatory compliance, and enable secure EHR exchange represents a promising future for blockchain within the healthcare sector as it continues to evolve and mature^5^.

### New applications

The current landscape in blockchain technology applications is characterized by a notable gap in the analysis of developed, assessed, and implemented solutions^17^. In recent years, there has been an essential change in the delivery of emergency medicine care, away from traditional healthcare models and towards the adoption of digital technologies, some examples of which include AI, ML and big data^18^. The variety, speed, and volume of personal health data have significantly increased due to the use of these technologies and the growth of healthcare services. The need for efficient data interchange across the ecosystem of healthcare provider companies has increased due to this rise. Big healthcare data holds great promise, but striking a delicate balance between authorized data applications and protecting patient security and privacy rights is a challenging task^19^. There has been a change in the healthcare sector to prioritize data protection and ownership^20^. Events like the most significant healthcare data breach of 2018, which exposed 13 million healthcare records and had severe repercussions, indicate that there is still some worry about the compromise of sensitive healthcare data^21^. In its most basic form, a blockchain is a distributed, decentralized ledger that facilitates transparent, safe record-keeping. The launch of blockchain technology version 1.0 and the launch of Bitcoin as a cryptocurrency occurred at the same time^22^. In particular, Blockchain 3.0 aims to give decentralized and trustless characteristics to a range of systems, especially in the healthcare industry^23^. Blockchain 3.0 expands the use of blockchain technology beyond financial transactions, whereas Blockchain 2.0 expands to distributed ledgers with smart contracts^24^. While there is a prevailing belief in the potential of blockchain to enhance health information systems, it is crucial to acknowledge that many ongoing discussions in this area are founded on inaccurate assumptions and misconceptions. To comprehensively assess the potential impact of blockchain technology within the healthcare domain, which encompasses healthcare, health sciences, and health education, it is imperative to delve into the current state of research in these respective fields. Such an analysis is essential to understanding how blockchain can be effectively leveraged to address critical challenges and improve various aspects of healthcare. Connected devices within the IoT play a pivotal role in data exchange thanks to their utilization of advanced, energy-efficient wireless communication channels^25^. These technologies offer heightened levels of security and utility, making them indispensable in healthcare settings. They serve multifaceted purposes, including identity verification, the correction of biometric data, data acquisition and analysis, and the reinforcement of security measures. These diminutive monitoring devices excel at collecting a plethora of physiological parameters, ranging from blood pressure, oxygen saturation levels, body temperature, heart rate, respiratory rate, levels of weariness, body weight, height, and even sleep duration, all while individuals are under observation. This wealth of data provides invaluable insights into patient health and well-being. Within the digital landscape, a specific segment is earmarked to facilitate patient care, and it goes by the moniker” the IoMT^26^. In the IoMT, various devices, including wheelchairs and beds, have been imbued with integrated electronic systems. This integration equips medical professionals, including physicians and nurses, with seamless access to patients’ biological data, enabling them to render accurate and prompt diagnoses. Because IoT devices generate enormous amounts of data that require rapid processing, portable devices and sensors are quite useful in this situation. Medical staff members are given more time when patients are admitted to hospitals to evaluate their conditions and decide whether or not life-saving procedures are necessary. Doctors and other participants must have an in-depth understanding of the interaction and correlation between numerous physiological features to provide patients with specific and optimal therapy^27^. As such, there is a lack of comprehensive research on the applications of blockchain technology in the healthcare sector, even if its potential is widely recognized. To properly utilize blockchain technology in the healthcare, health sciences, and health education domains, an in-depth examination of the current state of research is needed. The importance of innovative technology in modern healthcare environments is shown by the fact that IoT devices, which are a part of the Internet of Medical Things (IoMT), significantly contribute to data collection and healthcare improvement^23,28^. The term” Internet of Medical Things” (IoMT) refers to a broad category of products, including but not limited to beds and wheelchairs with built-in electronic systems^29^. These integrated systems are pivotal in modern healthcare settings, providing data and capabilities to enhance patient care. Within hospital environments, medical personnel, comprising physicians and nurses, are granted access to a treasure trove of patients’ biological data courtesy of the IoMT. This access empowers healthcare professionals to deliver accurate and timely diagnoses, ultimately leading to more effective treatment strategies. The ability to monitor patients’ vital signs and health parameters in real-time is a trans-formative aspect of IoMT. One of the defining characteristics of IoMT is the sheer volume of data generated by these integrated devices, and this data necessitates efficient processing and analysis^29^. IoMT portable devices and sensors excel in this regard, as they provide a means to capture, transmit, and interpret this data efficiently. This is particularly advantageous in healthcare contexts where quick decision-making and timely interventions can be life-saving. In cases where patients are referred to medical facilities, the IoMT offers medical professionals an extended time frame to evaluate the patient’s condition thoroughly. This extended duration is invaluable, allowing healthcare providers to consider various aspects of the patient’s health comprehensively. It enables them to explore potential life-preserving measures and tailored treatment plans, considering a holistic view of the patient’s health. The correlation between various health parameters, such as blood pressure, heart rate, oxygen saturation, and respiratory rate, is crucial for medical professionals and other stakeholders involved in patient care^29^. These correlations help healthcare teams make informed decisions and provide patients with optimized therapy regimens tailored to their needs. Furthermore, the IoMT’s capacity to provide many informational sources and the guidance of experienced medical professionals greatly facilitate the overall health management process for patients. It ensures that healthcare decisions are evidence-based, informed by real-time data, and guided by the expertise of trained professionals.

### Sensor-based technology

Importantly, sensor-based technologies integrated into the IoMT have the potential not only to monitor patients’ health but also to mitigate and, in specific circumstances, prevent potential medical crises. By continuously collecting and analyzing data, these technologies can identify anomalies and trigger alerts or interventions, ensuring that healthcare providers can respond promptly to emerging issues. Thus, IoMT is a transformative force in healthcare, encompassing various integrated devices that provide valuable data and capabilities. It grants medical professionals access to patient data, enabling accurate diagnoses and tailored treatments. The efficiency and extended evaluation time provided by IoMT devices are critical in healthcare decision-making. Additionally, the correlation between health parameters and the guidance of experienced professionals are essential components of effective patient care. Sensor-based technologies within the IoMT have the potential to not only monitor but also prevent medical crises, enhancing patient safety and well-being.

**Figure 1:**
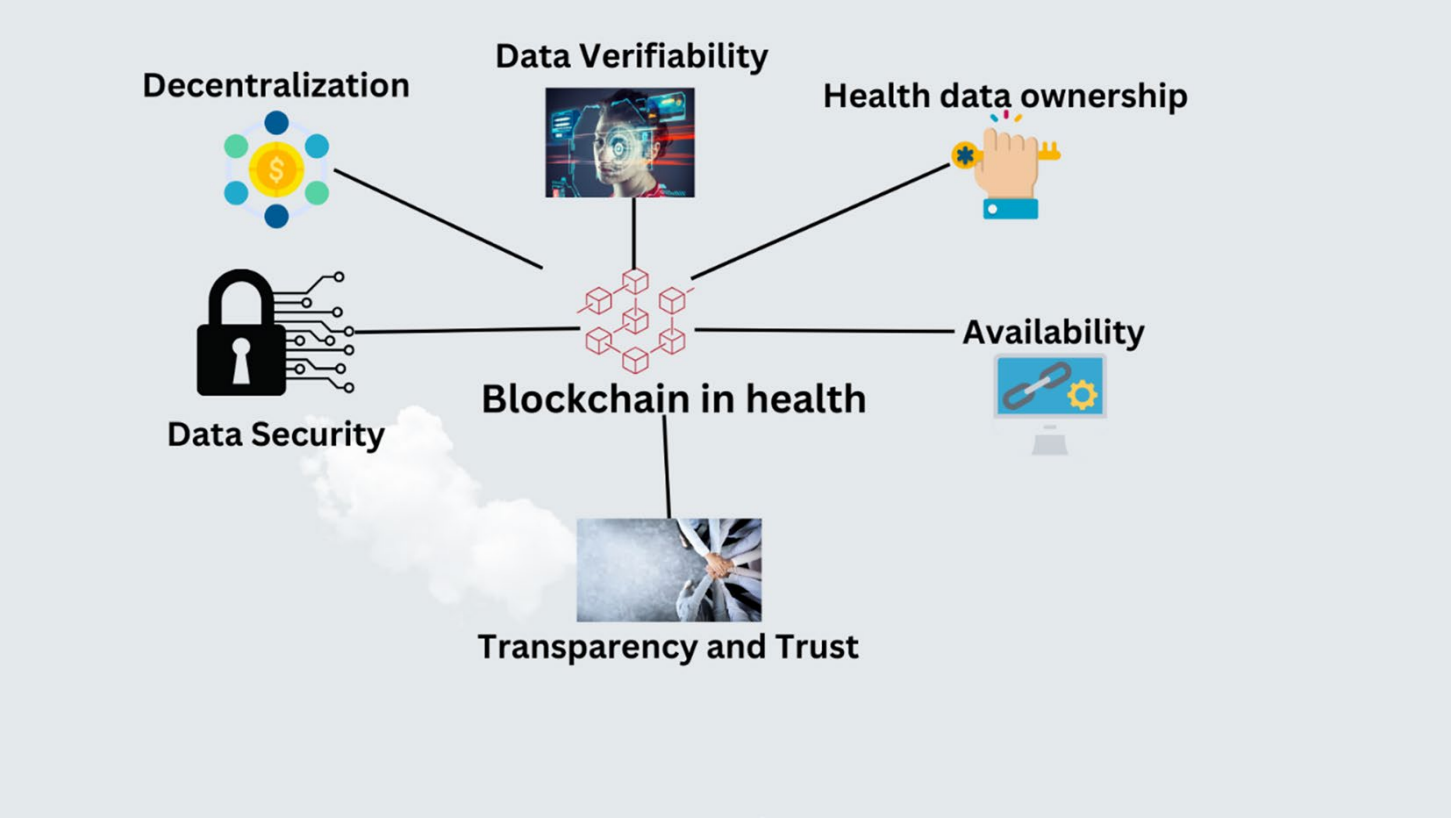
Advantages of Blockchain.

In recent years, blockchain technology has gained prominence as a reliable and decentralized platform, primarily attributed to its elimination of intermediaries and the inherent security mechanisms preventing unauthorized data alterations. When properly implemented, blockchain technology has the potential to enhance the management and security of IoT platforms significantly. Utilizing blockchain in healthcare, particularly in medical research and data management, holds promise for individuals, medical researchers, and healthcare professionals^30–32^. Figure 1 illustrates blockchain advantages in healthcare. One of the primary advantages of blockchain technology lies in its ability to provide a secure and transparent data storage and management platform. Within the healthcare domain, this feature is invaluable. Blockchain facilitates fast access to authorized individuals while safeguarding sensitive medical data. This security is especially critical given healthcare information’s stringent privacy and confidentiality requirements. Moreover, blockchain technology offers a distinct advantage in terms of accurate data visualization. During the initial phases of the COVID-19 pandemic, healthcare professionals faced the critical task of disseminating statistical data to inform public health measures and research efforts^33^. Blockchain’s role in this context was pivotal. Blockchain technology has accelerated research on the benefits and drawbacks of the pandemic by facilitating improved global connectivity and data availability. The decentralized nature of blockchain ensures that data remains tamper-proof and transparent, enabling healthcare professionals to trust the accuracy and integrity of the information they rely on for critical decision-making. This trust in data is essential in public health emergencies, as it influences the development of strategies, interventions, and policies. Furthermore, the utilization of blockchain technology in medical research has the potential to revolutionize the field. It can facilitate the secure sharing of research data among scientists and institutions, promoting collaboration while maintaining data integrity and privacy^31^. This collaborative research approach can expedite discoveries and advancements in healthcare. The results show that blockchain technology’s reliability, decentralization, and security features have made it a valuable asset in healthcare. Its capacity to securely store and manage medical data, facilitate data visualization, and support medical research holds substantial promise. Particularly in times of public health crises like the COVID-19 pandemic, blockchain’s role in data dissemination and research acceleration underscores its potential in shaping the future of healthcare. The accumulation of extensive datasets through global data collection presents a valuable opportunity for initiating research on various aspects of COVID-19^34^. However, this strategy must adhere to all relevant international laws and global standards to mitigate the risk of non-compliance with data-sharing rules.

### Motivations of the study

The motivations of the study outlined in this paragraph can be summarized as follows:

**A. Security and privacy concerns in IoT data transmission**

The study is motivated by the significant volume of data transmitted by sensor-based circuits and devices to IoT platforms. With the internet being the medium for data transmission, concerns regarding the security of personal data and data privacy have emerged as crucial issues. Additionally, the speed of data transmission over networks is a concern that needs attention. The study recognizes the need to address these concerns before allowing unrestricted data transmission on internet-connected devices.

**B. Blockchain as a solution**

The study is motivated by the potential of blockchain technology to address these security and privacy challenges. Blockchain offers a decentralized and confidential environment for data utilization, making it a practical approach to enhancing data security and privacy.

**C. IoT scalability and efficiency**

The study acknowledges the advancements in the IoT ecosystem, particularly in addressing challenges related to scalability, latency, and processing efficiency through edge-fog-cloud computing. It aims to explore how blockchain technology can further enhance security in this context.

**D. Enhancing privacy and trust in healthcare**

The study references a comparative analysis of research gaps in secure communication among IoT-connected devices. It recognizes the potential of blockchain technology to enhance privacy, trust, and authentication within operational healthcare devices. Ensuring the protection of user privacy is a crucial motivation.

**E. Challenges in data acquisition via IoMT**

The study acknowledges challenges related to data acquisition in the IoMT due to the increasing number of low-power devices with constrained data transmission capabilities. It suggests that advancements such as Low-Power Wide-Area Networks (LP-WANs) and 5G may help address these challenges.

**F. Exponential growth of medical data**

With a prediction of exponential growth in medical data generated by devices, the study anticipates data storage and replication challenges. It emphasizes the need for secure data transmission and storage, distinguishing between crucial and less crucial data.

**G. Complexity of healthcare data control**

The study recognizes the sensitive nature of healthcare data and the extensive network of medical establishments. It suggests that governmental measures may be necessary to ensure proper control and governance of healthcare data.

**H. Pertinent research aspects**

As a researcher, the study suggests investigating several aspects of medical data, including regulations and privacy implications, challenges in data sharing, and scalability issues in healthcare facilities.

**I. Addressing pandemics**

The study identifies research paths to manage COVID-19 effectively, emphasizing the importance of distributed systems and smart city environments.

In a few words, the motivations of the study revolve around addressing security and privacy concerns in IoT data transmission, exploring blockchain as a solution, enhancing the efficiency and scalability of IoT, improving privacy and trust in healthcare, and addressing challenges associated with the exponential growth of medical data and data control in healthcare settings. It also emphasizes the relevance of research in the context of pandemics and the potential of distributed systems in smart city environments.

### Contributions of the study

This comprehensive review of blockchain technology covers all of its aspects, elements, and protocols. This article examines the critical role that blockchain technology plays in the IoMT, exploring further the mutually beneficial relationship between blockchain and the IoT. By analyzing many examples and applications that illustrate the use of blockchain technology, the research aims to provide an in-depth comprehension of the collaborative use of blockchain technology in IoMT. The study carefully analyses and examines the many difficulties that IoMT systems face within the context of this blockchain integration. This explains the complexity of the problems at issue as well as the interaction between blockchain technology and the IoT, highlighting their deep complexity. Beyond just mentioning these difficulties, the study offers several approaches to solving them. As such, it makes a substantial contribution to our understanding.

This study also focuses on investigating blockchain technology’s possible medical applications. In this paper, we examine several practical examples that show the successful application of blockchain technology to the IoMT and healthcare. The study results are presented in an organized and thorough manner by grouping these contributions into three major categories. The fact that this organizing strategy facilitates comprehension of the data and insights highlights the study’s contributions to the discipline.

### Organization of paper

The remaining parts of the article are structured as described below. A brief description of blockchain and IoT is covered in Section "Literature review". Section "Methods and materials" discusses the technologies that enable secure and advanced IoT-based medical applications. Section "Results" describes the Blockchain and IoT integration for developing advanced IoMT applications. The future open challenges are described in “Open challenges to the integration of blockchain with the IoMT”. Section “Solution of key challenges” describes the conclusion. Table 1 illustrates a list of abbreviations.

**Table 1:** List of abbreviations.

Abbreviations	Description	Abbreviations	Description
IoT	Internet of things	AI	Artificial intelligence
IMOT	Internet of medical things	PoW	proof-of-work
PoS	proof-of-stake	P2P	peer-to-peer
MITN	Man-in-the-middle	PHI	Protected health information
SAT	Security access token	EHR	Electronic health records

## Literature review

### Blockchain technologies in healthcare

In the context of healthcare and medical data, maintaining the privacy rights of individuals regarding their confidential medical information is paramount. Such sensitive data must be released with meticulous care and attention to ethical and legal considerations. Oversight and regulation are provided by frameworks such as the Health Insurance Portability and Accountability Act of 1996 (HIPAA) in the United States^35^ and analogous international data protection laws. These rules aim to safeguard patient privacy while facilitating the authorized and secure sharing of data that is essential to the healthcare system. Understanding these rules is essential to ensuring that people’s medical records are not accessed or disclosed without authorization, protecting the privacy of their personal health information^32^.

In parallel, emerging concepts outlined in literature, such as reference^35^, offer innovative ways to facilitate convenient and secure user information sharing. These concepts are particularly relevant in healthcare and IoT medical devices. The utilization of IoT medical devices for monitoring a patient’s health has witnessed a growing trend, becoming increasingly common in healthcare settings. These devices, characterized by their ability to provide real-time data on physiological parameters, offer a straightforward assessment of an individual’s health status. This includes parameters like temperature, oxygen saturation, heart rate, and the capability to assess internal body temperature^36^.

### Enhancing security

The use of blockchain technology enhances the security and privacy of the data produced by Internet of Things (IoT) healthcare devices. It guarantees the privacy of sensitive health information while granting authorized parties access to relevant information when needed. This secure and transparent data management technique is by the principles of patient privacy and data protection supported by legal frameworks like HIPAA^37,38^. Thus, collecting extensive datasets for COVID-19 research necessitates strict adherence to international laws and standards to protect patient privacy and data security. Regulatory frameworks like HIPAA play a crucial role in overseeing the release of medical records. Innovative concepts in information sharing, as outlined in Jerbi et al.^36^ further advance secure data-sharing practices. Meanwhile, the increasing prevalence of IoT medical devices underscores the importance of robust data security, which can be achieved through blockchain technology integration.

Utilizing blockchain technology in conjunction with IoMT devices holds significant potential for enhancing patient privacy protections and optimizing the functionality of these devices^30^. This integration offers numerous advantages rooted in blockchain technology’s decentralized and secure nature. One of the key benefits of combining blockchain and IoMT is the bolstering of patient privacy. Blockchain’s decentralized architecture ensures that sensitive health data remains secure and tamper-proof. Patient records and data can be stored on the blockchain so only authorized individuals or entities can access them. This enhances patient confidentiality and data security, aligning with regulatory requirements and ethical considerations. Furthermore, eliminating centralized intermediaries is a pivotal aspect of blockchain technology. Traditional healthcare systems often involve multiple centralized entities and organizations responsible for data management. The integration of blockchain eliminates the need for these intermediaries, streamlining the process of transmitting patient data and information across global platforms^30^. This not only enhances data accessibility but also reduces the risk of data breaches and unauthorized access.

In a broader context, blockchain technology promotes enhanced and decentralized communication of data and information between healthcare facilities, professionals, and patients. Hospitals and healthcare providers can securely share patient records, test results, and treatment plans with patients, ensuring transparency and fostering trust in the healthcare ecosystem. Integrating blockchain with IoT-enabled devices further facilitates the seamless distribution of a patient’s medical records to individuals worldwide. This means authorized parties can access relevant medical information securely and efficiently, regardless of geographical location. This is particularly valuable in emergency medical care or remote consultations, where quick access to accurate medical data is critical. A vital component of blockchain technology in IoMT is using reliable smart contracts to transfer IoMT data to the blockchain. Smart contracts automate and enforce predefined rules, ensuring data integrity and authenticity. This enhances the system’s resilience against data manipulation and fabrication, safeguarding the accuracy of medical records and treatment histories.

The inherent security features of blockchain, including cryptographic encryption and decentralized consensus mechanisms, contribute to establishing mutual trust among all participating parties in the healthcare ecosystem^30^. Patients can trust that their data is protected, while healthcare providers can rely on the integrity of the information they access.

Alsemmeari et al.^39^ focus on the implementation of a comprehensive evaluation system designed to analyze web-based healthcare apps effectively. The authors used the AHP and TOPSIS methods to compare and rank different options based on things like data integrity, auditing standards, resilience, authentication, encryption, and the ability to revoke access. Particularly, our research is consistent with the computational methodology suggested by Ahmad et al.^40^ to perform an empirical inquiry into the best security practices for medical equipment. Similarly to that, the subsequent study employed AHP, hesitant fuzzy, and AHP techniques to assess several options related to criteria characteristics. Passwords, version control, software recovery, access control, biometric authentication, security tokens, backups, and error detection were among these features. In this subject, the work of Alsemmeari et al.^39^ is regarded as unique. To improve the security of IoMT, they developed a robust architecture in their study that utilizes TNN and blockchain technologies. In contrast, this paper offers a brand-new framework that uniquely combines the core features and advantages of blockchain technology with artificial intelligence. To increase the number of IoT devices, IoMT systems will be provided with an automated process. This methodology aids in both the detection of ongoing cyberattacks and the process of learning from them to anticipate and predict new threats.

### Decentralized storage

Additionally, blockchain’s decentralized storage concept simplifies and enhances transparency in healthcare data management, including data collection, sharing, and storage^10,11,41,42^. It ensures that data is stored securely and can be audited for accuracy and compliance. Integrating blockchain technology with IoMT devices results in robust patient privacy protection, streamlined data transmission, enhanced communication, and increased trust within the healthcare ecosystem. Its decentralized and secure nature aligns with regulatory requirements and ensures the integrity and confidentiality of medical data.

Most cloud systems are typically hosted in large, centralized data centers physically distinct from the Internet’s core infrastructure. These data centers play a crucial role in storing and managing the vast volumes of data generated by interactions between (IoT) devices and their surrounding environments. Storing data on centralized computers^43^ has been a common practice, but it introduces vulnerabilities, notably when adequate security measures are lacking. Unauthorized individuals may exploit security weaknesses to gain unauthorized access to data stored in specific locations^44^. Moreover, user data is often distributed across geographically distant data centers, a strategy intended to enhance data redundancy and availability. However, the geographical placement of data centers and their immediate surroundings can significantly impact the delivery of services to residents in a particular region. Therefore, ensuring optimal performance and effective administration of data centers is imperative in this context.

To address the challenges outlined in this research, peripheral computing and fog computing have emerged as potential solutions. These enhancements present an opportunity to improve system bandwidth and reduce latency, critical factors for efficient data processing and communication. Furthermore, peripheral and fog computing have evolved into essential tools for performing supplementary activities within the IoT ecosystem. A comparative analysis conducted in a study^45^ highlighted research gaps arising from the failure of IoT-connected devices to establish secure communication channels among themselves. While the authors expressed their intention to adhere to established procedures, they recognized the potential for enhanced security by implementing multiple layers of blockchain technology. This approach aimed to enhance privacy, trust, and authentication within the operational devices of the healthcare system. However, it is essential to note that the precise outcomes of this model remain uncertain as it has not yet been tested in real-world scenarios.

### Acquiring data

Acquiring data via the IoMT presents challenges^46^, primarily due to the proliferation of low-power devices with constrained data transmission and reception capabilities. However, there is optimism regarding resolving this issue with rapidly deploying technologies such as low-power wide area networks (LP-WANs) and 5G^47^. These advancements can potentially improve data acquisition and communication in IoMT, thereby enhancing the overall functionality of healthcare systems.

The centralized nature of cloud systems and security concerns motivate the exploration of alternative computing paradigms like peripheral and fog computing. Additionally, the integration of blockchain technology holds promise for enhancing security and trust within IoT-connected devices, particularly in healthcare applications. Finally, advancements in network technologies like LP-WANs and 5G offer solutions to data acquisition and transmission challenges in IoMT environments. These employment initiatives have utilized the authors’ conceptual framework, known as BCeMT, in the context of disease prevention. The objectives of the BceMT architecture are enhanced interoperability and user privacy protection. The framework employs bitwise XOR and cryptographic hash methods to achieve data security goals. Furthermore, a comprehensive framework for integrating blockchain technology into current medical contexts has been provided by A related study by Ahmed et al.^48^ introduced a sensor-based architectural design for an urban healthcare center operating autonomously. This innovative design incorporates artificial intelligence (AI) and IoT technologies, marking a significant advancement in healthcare infrastructure. Additionally, Kumari et al.^49^ proposes incorporating a new blockchain layer into the IoMT architecture. IoMT comprises three distinct yet interconnected components: medical services, data management, and sensing. The responsibility for making related decisions lies within each of these hierarchical levels. BceMT is introduced as a supplementary component to the standard three phases of IoMT. Its primary objective is to support the uppermost three layers within the existing IoMT stack, ensuring privacy in all interactions^50^. Anticipating exponential growth in data generated by medical devices, implementing the proposed architecture presents several challenges. Limited accessibility to medical devices remains a significant obstacle for individuals seeking healthcare services. Data replication is distributed across multiple peers within a blockchain network, contrasting with the centralized approach commonly employed. This decentralization poses an additional challenge in the context of blockchain technology implementation.

Consequently, selecting a cloud storage service with substantial data storage capacity becomes imperative. Blockchain technology is leveraged to securely transmit critical data, while less crucial data can be stored on cloud servers, effectively reducing the workload on network nodes. The complexity of healthcare data management is compounded by the sensitive nature of the data and the extensive network of hospitals and medical establishments involved. This complexity underscores the importance of implementing governmental measures. As a researcher, one may consider investigating various critical aspects related to medical data, including (I) the imperative of regulations and associated privacy implications, (II) the challenges of sharing medical data without assurances against mishandling or unauthorized disclosure, and (III) the issue of scalability, given the potential rapid expansion of network dimensions leading to a substantial influx of data within already congested healthcare facilities^51^.

Furthermore, the study identifies multiple research paths with the potential to address pandemics like COVID-19 effectively. The researchers have also developed a distributed system to manage IoT devices in smart city environments. This system is structured into three distinct layers: the Energy Generation and Distribution Layer, the Consumer-Producer Layer, and the Communication Layer. Presenting opportunities for innovative solutions in smart city infrastructure^52^. Healthcare technology improves patient care, enhances efficiency, and reduces costs. Healthcare technology aims to support providers in delivering better and more personalized patient care while improving the overall healthcare system^53^.

### Technological advancement

The advancement in technology has led to significant developments in e-health. E-health comprises electronic health records, telemedicine, mobile health apps, and wearable devices to improve healthcare delivery and patient outcomes^54^. Patients can now receive remote medical consultations and diagnoses through video conferencing and remote monitoring tools. These technologies provide patients with tools to monitor and manage their health, including tracking vital signs, medication adherence, and fitness levels^55^. The role of the IoT, machine learning, and blockchain in e-health is significant and growing. IoT refers to the network of physical objects, devices, vehicles, buildings, and other items embedded with sensors, software, and connectivity, allowing them to collect and exchange data over the Internet. IoT enables these objects to communicate with each other and central servers, allowing for real-time monitoring, control, and automation of various processes and systems^56^.

This technology has the potential to revolutionize industries and improve efficiency, convenience, and quality of life for individuals and communities. Blockchain is a digital ledger technology that enables secure, transparent, decentralized transactions and data management. It is a distributed database that uses cryptographic methods to ensure that each link in the chain is unchangeable and cannot be changed in the past without the network’s permission^57^. A chain that has a complete record of all the data and transactions on the network is created when blocks are joined to one another in chronological order. With this technology, peer-to-peer transfers can occur without the need for trust, eliminating the need for centralized authority to verify and authenticate transactions^58^. It has the power to completely change the financial, supply chain, and healthcare industries. Blockchain technology holds promise for enhancing supply chain management, enabling secure transactions, and securely storing and exchanging patient data inside the electronic health domain^59^.

IoT and blockchain can enable more efficient, personalized, and secure e-health solutions to improve patient outcomes and enhance the healthcare system^60^. Blockchain can solve various challenges in I.T. by providing a decentralized, fast, and transparent way of storing and sharing data. Blockchain can enable different systems and applications to share data and communicate with each other through smart contracts^61^. IoT and Blockchain technology can provide a secure means to manage and transfer data in the IoMT systems^62^. Blockchain can handle big data in several ways by storing data distributed across a network of nodes, which can help alleviate the storage burden on a single server or database^63^. Blockchain-based smart contracts can automate processes and enable automatic data transfers and payments, reducing the need for human intervention and streamlining data management processes blockchain technology can allow a more transparent and secure way to manage medical data, which can help to build trust and improve patient outcomes in e-health^64^.

**Figure 2:**
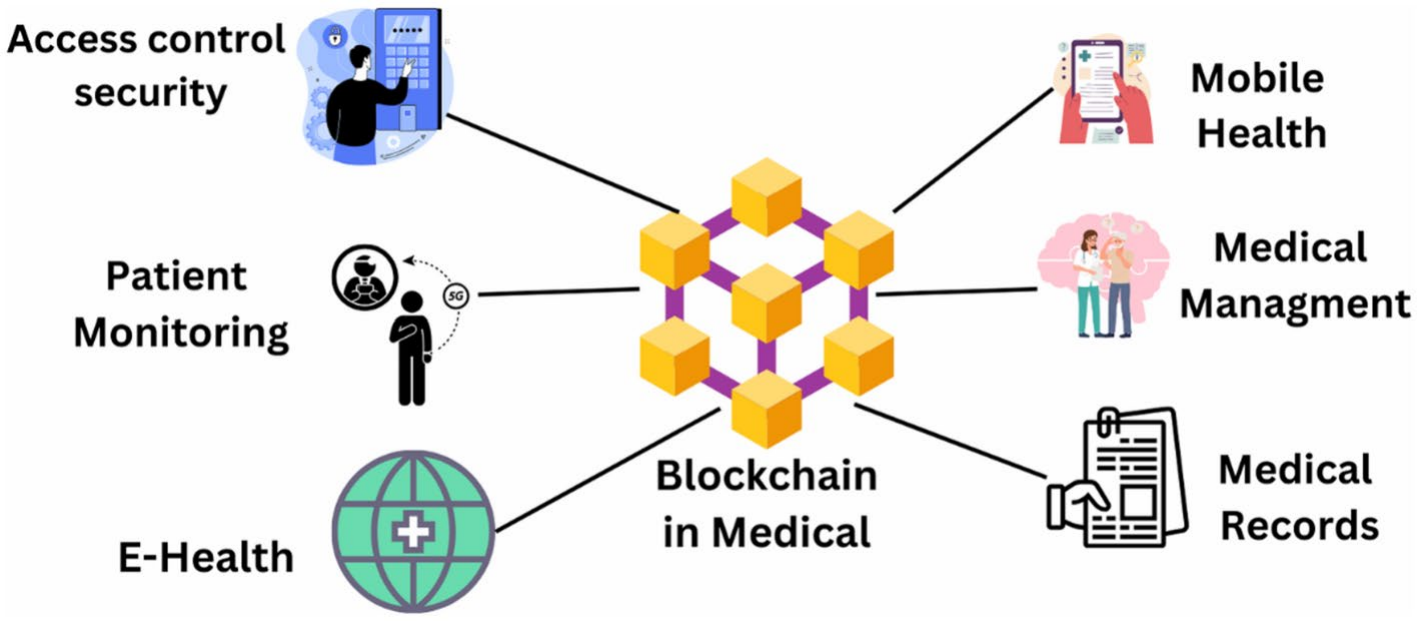
Application of Blockchain in healthcare.

Blockchain technology can enable authorized parties, such as doctors and healthcare providers, to access and share medical data securely and transparently. The integration of blockchain and IoT can improve security by preventing unauthorized access, interference, and data breaches due to blockchain’s decentralized and cryptographic nature^65^. It ensures data integrity because the data stored on a blockchain is unchanging and cannot be changed. Blockchain resolves several IoT-related issues, yet it faces numerous challenges. Blockchain and IoT create massive volumes of data, and combining them can cause scalability issues^66^. The objective is to create a blockchain system that can manage many transactions quickly and efficiently. Implementing blockchain with IoT could be expensive due to the significant investment required in infrastructure and the creation of new systems and applications^67^. There are no clear regulations governing the usage of blockchain and IoT, making it challenging to adopt these technologies in a compliant way^68^. IoMT has medical devices, sensors, and other medical equipment connected via the network. These devices can communicate with each other to collect and transmit medical data, as mentioned in Fig. 2.

### Benefits of blockchain

IoMT devices can increase the risk of cybersecurity breaches and data privacy concerns, as these devices collect and transmit sensitive medical data. It is essential to implement robust security measures to protect the devices and the data they collect^69^. Table 2 illustrates the advantages of blockchain in healthcare. Blockchain has emerged as the most reliable decentralized platform due to its ability to facilitate transactions without an agent and its strict guidelines against data modification, which safeguard private information. IoT platforms might have a lot less security and management problems if blockchain technology is used^70^. People, medical researchers, and healthcare providers can all benefit from developing a website that keeps track of personal information, records health data, and offers users dependable access to data^71^. By enabling correct data viewing, blockchain technology also provides this benefit. Information exchange between healthcare entities was one of the most significant responsibilities during the global COVID-19 outbreak. Research regarding the benefits and drawbacks of the epidemic may now be conducted more quickly due to the increased worldwide connectivity and data available. A wide range of COVID-19 research challenges can be investigated and resolved using high-level sources from global data collection. Ensuring compliance with all foreign laws and standards is vital to preventing potential violations of data-sharing laws.

**Table 2:** Benefits of blockchain to healthcare applications.

Advantages	Description
Decentralization	Implementing a decentralized management system is crucial to the healthcare sector’s overall performance. Blockchain technology applications help with decentralized health data administration. In a safe and free manner, this would enable access to a patient’s medical records for all parties involved in their care
Improved data security and privacy	Once data is stored on a blockchain, it becomes unchangeable, making it impervious to alterations, deletions, or corruption. The records are appended in reverse sequential order, with the present time also inserted. Additionally, all medical information is encrypted using blockchain technology. In addition, it should be noted that patients’ medical data undergoes encryption through the utilization of cryptographic keys before its storage on the blockchain. Limiting the probability of identity theft and safeguarding patient confidentiality are outcomes that can be achieved using this measure
Health data ownership	All patients should have unlimited access to their health records so they can make choices about their care. People who are ill require assurances that their medical records will be kept private and that they will be quickly notified if something happens to them. A basic right of patients is the ability to view and assess their medical records. Blockchain technology, which combines open smart contracts and reliable cryptography, makes this possible
Availability/robustness	Utilizing blockchain technology ensures the continuous accessibility of medical records stored on the ledger, as it possesses robust resistance against data loss, corruption, and specific data availability security breaches. This phenomenon arises due to the replication of blockchain data over numerous computers, commonly called “nodes”
Transparency and trust	Due to its inherent transparency, blockchain technology has the potential to enhance the reliability of decentralized applications within the healthcare industry. Consequently, more individuals employed within the healthcare sector will likely exhibit a greater receptiveness toward utilizing these applications
Data verifiability	Data saved on a blockchain cannot be changed in any way, shape, or form, even if the original plaintext records are unavailable. Healthcare claim processing and pharmaceutical supply line management are two critical areas that require record evaluation. Important are both of these domains. These areas now get significantly more benefits than in the past, due to this knowledge

Global communication becomes more natural for everyone, and patients have greater freedom when there is no longer a requirement for a central server or for authorities to stand between patients and their data. Blockchain technology generally gives people more power. It becomes possible for hospitals and patients to communicate effectively and decentralize relevant data and information^72^. A patient’s medical history can be easily reconstructed using blockchain technology and IoT, provided that a worldwide deployment strategy for IoMT-powered devices is put into place, safely moving information to a blockchain from the IOMT. The system that gathers, transfers, and saves data is also made incredibly easy to maintain and open for inspection by anybody by utilizing a blockchain^43^.

The IoT and blockchain have many applications in the medical field. This research provides a comprehensive overview of Blockchain and IoT to help readers understand the fundamentals of Blockchain and IoT related to medical applications. Table 3 illustrates the analysis of some previous studies.

**Table 3:** Analyses of this research in comparison to previously published survey studies.

SR	Difficulties and solutions in IoMT	IoT	Blockchain	Solutions protected IoMT	Reference
1	X	✓	✓	✓	Kashani et al.^73^
2	X	✓	✓	X	Uddin et al.^74^
3	X	✓	X	X	Sworna et al.^75^
4	✓	✓	X	✓	Karthick and Pankajavalli^76^
5	X	X	X	X	Qayyum et al.^77^
6	✓	✓	✓	X	Qadri et al.^79^
7	✓	✓	X	X	Wang et al.^80^
8	✓	✓	✓	X	Zaman et al.^82^
9	X	X	✓	X	Andoni et al.^81^
10	✓	✓	X	X	Aggarwal et al.^85^
11	X	X	X	X	Shailaja et al.^78^
12	✓	✓	✓	X	Panarello et al.^83^
13	X	✓	X	X	Faust et al.^87^
14	X	X	✓	X	Kuo et al.^84^
15	X	✓	X	X	Ahmadi et al.^86^

As recapitulated in Table 3, our study stands out by addressing the specific security and privacy challenges faced by the IoMT through the integration of blockchain technology with IoT. Unlike Kashani et al.^73^, who focus on general security enhancements through blockchain-IoT integration, our research introduces additional layers of data protection tailored for medical applications, ensuring compliance with HIPAA standards. Similarly, while Uddin et al.^74^ explore blockchain in IoT, they do not delve into IoMT-specific issues. Our study addresses this gap by providing a detailed analysis of IoMT-specific problems, such as data integrity and patient privacy, and offers practical solutions like decentralized storage and smart contracts for secure data management.

In comparison to other works, our study extends beyond the scope of Sworna et al.^75^ and Karthick and Pankajavalli^76^, who either focus solely on IoT or do not integrate blockchain. We enhance their findings by incorporating blockchain to tackle IoMT-specific security and data management issues, providing a more robust solution. Unlike Qayyum et al.^77^ and Shailaja et al.^78^, who do not address IoMT, IoT, or blockchain, our research offers a comprehensive analysis and practical solutions to enhance IoMT security and privacy. Additionally, while Qadri et al.^79^, Wang et al.^80^, and Andoni et al.^81^ discuss aspects of blockchain and IoT integration, they lack specific solutions for IoMT protection. Our study fills this gap by offering detailed strategies such as smart contracts and decentralized storage systems.

Moreover, our research expands on the findings of Zaman et al.^82^, Panarello et al.^83^, and Kuo et al.^84^, who provide thorough analyses of IoT and blockchain but do not focus on IoMT protection. We ensure patient data privacy and compliance with healthcare regulations through specific IoMT security solutions. The work of Aggarwal et al.^85^ and Ahmadi et al.^86^, which focuses on IoT without blockchain, is further developed upon in our study. We integrate blockchain to address the challenges of IoMT and provide a data management system that is both secure and efficient. Additionally, we build upon the foundational work of Faust et al.^87^ by integrating blockchain to offer enhanced security and data management solutions for IoMT. This showcases the comprehensive nature and practical applicability of our research.

## Methods and materials

### Research questions

**Figure 3:**
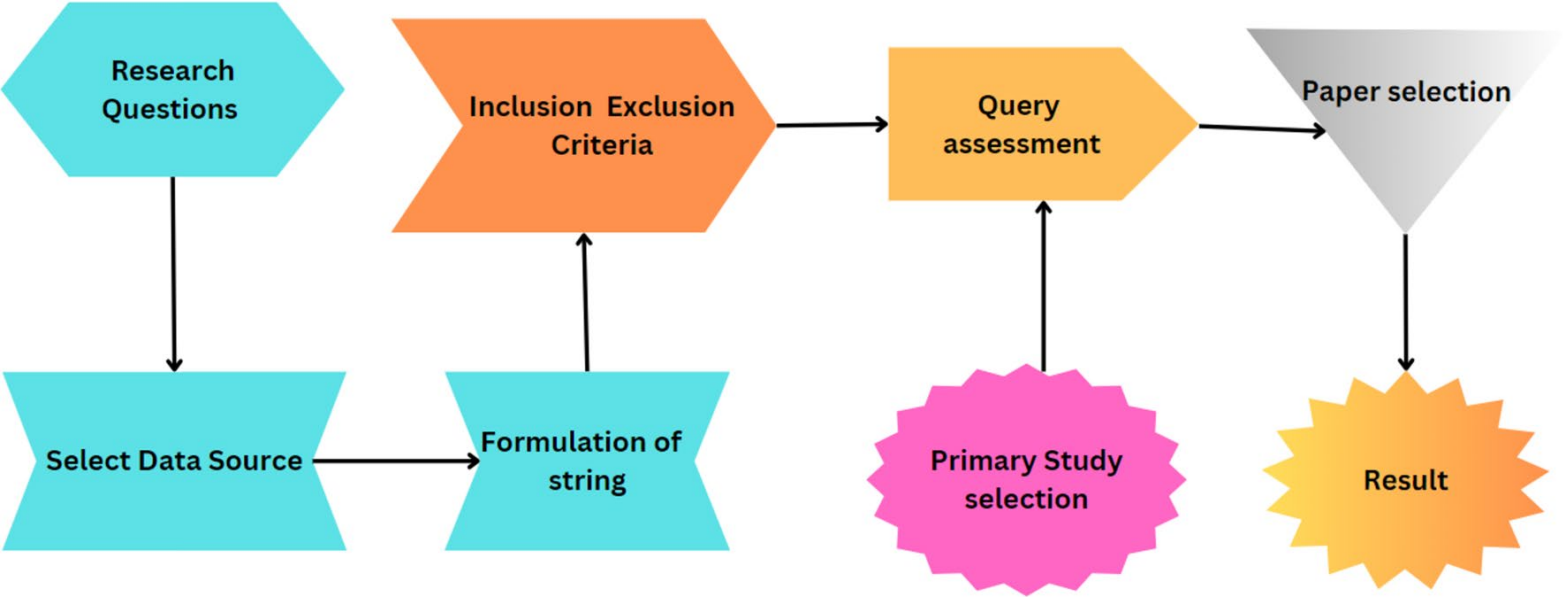
Flow of study.

The primary objective of this study is to conduct an SLR that identifies, analyzes, and summarizes empirical evidence related to the integration of blockchain with IoMT and the application of blockchain. The review focuses on using blockchain in the medical field and others. It also focuses on issues and their solutions to integrate blockchain with IoMT. Furthermore, it also focuses on blockchain types, components, architectures, protocols, and devices, with some case studies. It also focuses on the architecture of IoMT and its advantages. The research questions and the motivation behind each question have been formulated to guide the review process to achieve this goal. Table 4 illustrates the research question, and Fig. 3 shows the flow of the study.

**Table 4:** Research questions.

Research questions	Motivation
What are the applications, components, and types of blockchain	To know about the complete architecture of blockchain
What are the challenges in IoT-based medical systems	To know about the different types of challenges of IoT-based systems in healthcare
What are the different types of IoT-based medical applications	To know about the other IoT applications in medical
What is the Integration of Blockchain with IoT applications to develop a secure IOMT	To know about the development of secure IOMT using blockchain and IoT
What are the different types of applications of blockchain in medical health care	To know about the use of blockchain in medical healthcare
What are the different types of case studies of blockchain in IOMT	To know about different types of case studies of blockchain in IOMT
What are the Key challenges with the integration of blockchain with IOMT	To know about the challenges related to the integration of blockchain with IOMT
RQ7: What are the solutions to Key challenges of integration blockchain with IOMT	To know about the solution to these challenges related to blockchain integration with IOMT

### Select data sources

**Figure 4:**
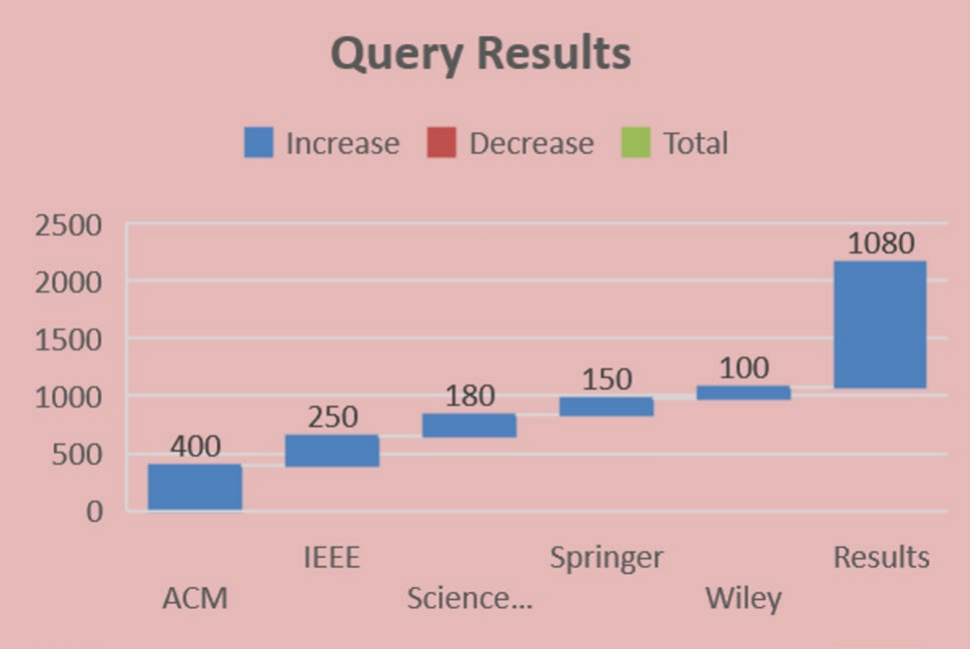
Query results.

Data sources are the libraries or repositories from which the research studies should be retrieved. Four digital libraries have been chosen to extract the primary analyses: IEEE Explore, Science Direct, ACM Digital Library, and Springer Link^88^. The full text of the documents is searched to identify the prior studies. There are various options available to search each digital library for pertinent information. To find the most relevant literature, the search strategy is modified to satisfy the needs of the respective data source. Selected data sources and the number of studies produced by search queries are illustrated in Table 5 and Fig. 4.

**Table 5:** Query results from data sources.

Library	Initial	Title and keyword	Abstract	Full text
IEEE Xplore	250	200	90	50
Science Direct	180	110	75	35
ACM Digital Library	400	250	130	80
Springer Link	150	120	70	40
Wiley	100	80	45	16
Result	1080	760	410	221

An SLR refers to a technique employed in locating, appraising, and synthesizing research evidence on a selected topic systematically and with high replicability to reduce biasing factors that might affect the findings' reliability

Initial: We begin with the total number of studies identified through database searches.

Title and Keyword: The number of studies remaining after screening titles and keywords for relevance.

Abstract: The subset of studies was further refined after reviewing abstracts for more detailed relevance to our research questions.

Full Text: The final count of studies included in our review after full-text evaluation confirmed their relevance and contribution to the field.

These categories play a vital role in portraying the filtration process in an SLR where studies are successively sieved down to those most germane to the research queries. A delineation such as this, laid out step-by-step, not only paints a vivid picture for readers on how we arrived at our final sample but also ensures transparency laced with clarity in enunciating our research methodology.

### Search string formulation

**Figure 5:**
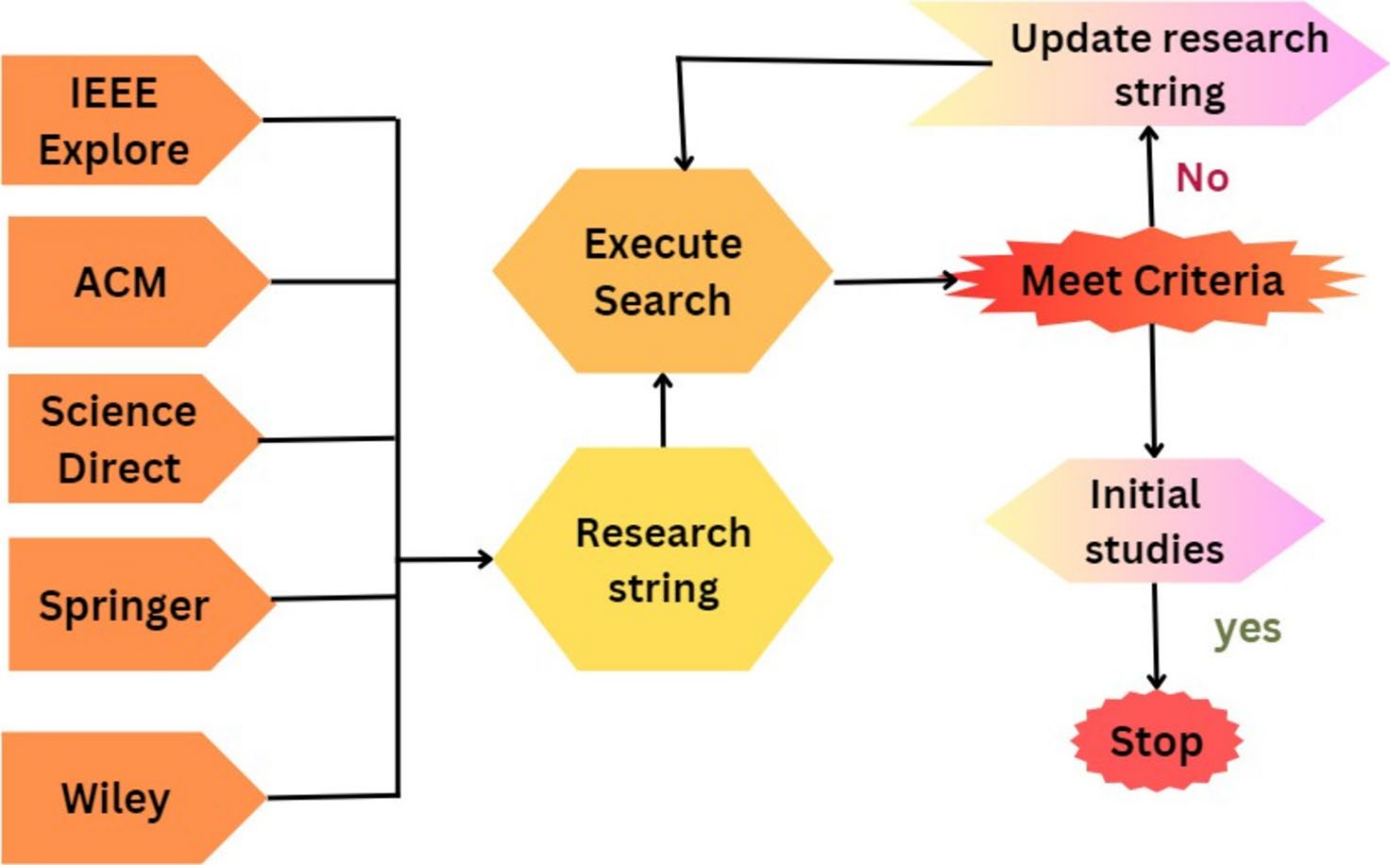
The process of formulating the search string.

A search string is a carefully crafted combination of keywords and search operators used to identify relevant studies that address the research question or topic of the review. This step focuses on specific keywords and their synonyms chosen from the identified research questions, as indicated in Table 4, to create the search string. These keywords are put together using the ’AND ’OR’ conditions in the order listed to complete the following search string: Fig. 5 and Table 6 illustrate the process of formulating a search string.

**Table 6:** Keyword selection.

Primary keywords	Synonym/alternative word
Blockchain	(“blockchain” OR “blockchain”)
Health	(“Healthcare ” OR “Medical Healthcare ”)
Application	(“metrics” OR “classification”)
Methods	(“Techniques ” OR “Framework ”)
Integration	(“Combination,” “OR “merge ”)
IoMT	(“IoT” OR “IoT medical”)

### Inclusion and exclusion criteria

Inclusion criteria in an SLR (Systematic Literature Review) refer to the predefined rules used to determine which studies would be included in the review. In this review, the following inclusion criteria will be considered:

- Studies must have been published in the English language within the timeframe of 2016 to 2023
- The subject of the study should be centered on blockchain utilized in the healthcare domain.
- Selected studies must involve empirical research, conducting practical experiments on specific datasets.
- The investigations undertaken in the study should pertain to the applications, architecture, and components of blockchain and IoMT.
- Each chosen study must encompass a comprehensive evaluation of blockchain and integration of blockchain with IoMT.
- The subject of the study should be centered on issues and their solutions during the integration of blockchain and IoMT.
- The scope of selected articles should be confined to publications in reputable journals, conferences, or books.
- Exclusion criteria in an SLR refer to pre-designed conditions to determine which studies will be excluded from the review.

The following categories of studies have been designated for exclusion:

- Those published before 2016.
- Those primarily focus not on blockchain and its application in healthcare.
- Studies that lack empirical analysis results.
- Studies that fail to evaluate the performance of blockchain and IoMT.

### Define quality assessment criteria

Quality assessment criteria in an SLR refer to the predefined standards or guidelines used to assess the included studies’ quality, reliability, and validity. Defining quality assessment criteria ensures that the selected primary studies offer sufficient details to analyze the identified research question effectively. In this step, a standard is defined for each research question. Each quality assessment criterion is denoted by C and its respective number, as shown in Table 7.

**Table 7:** Quality assessment criteria.

Quality assessment	Questions
C1	Does the study provide enough information about the history of blockchain?
C2	Does the study provide enough information about the integration process of blockchain with IoMT?
C3	Does the study provide enough information about blockchain and its role in HER?
C4	Does the study provide enough information about the application of blockchain and some case studies in healthcare?
C5	Does the study provide enough information about challenges and their solutions in the integration process of blockchain with IoMT?

### Primary study selection

**Figure 6:**
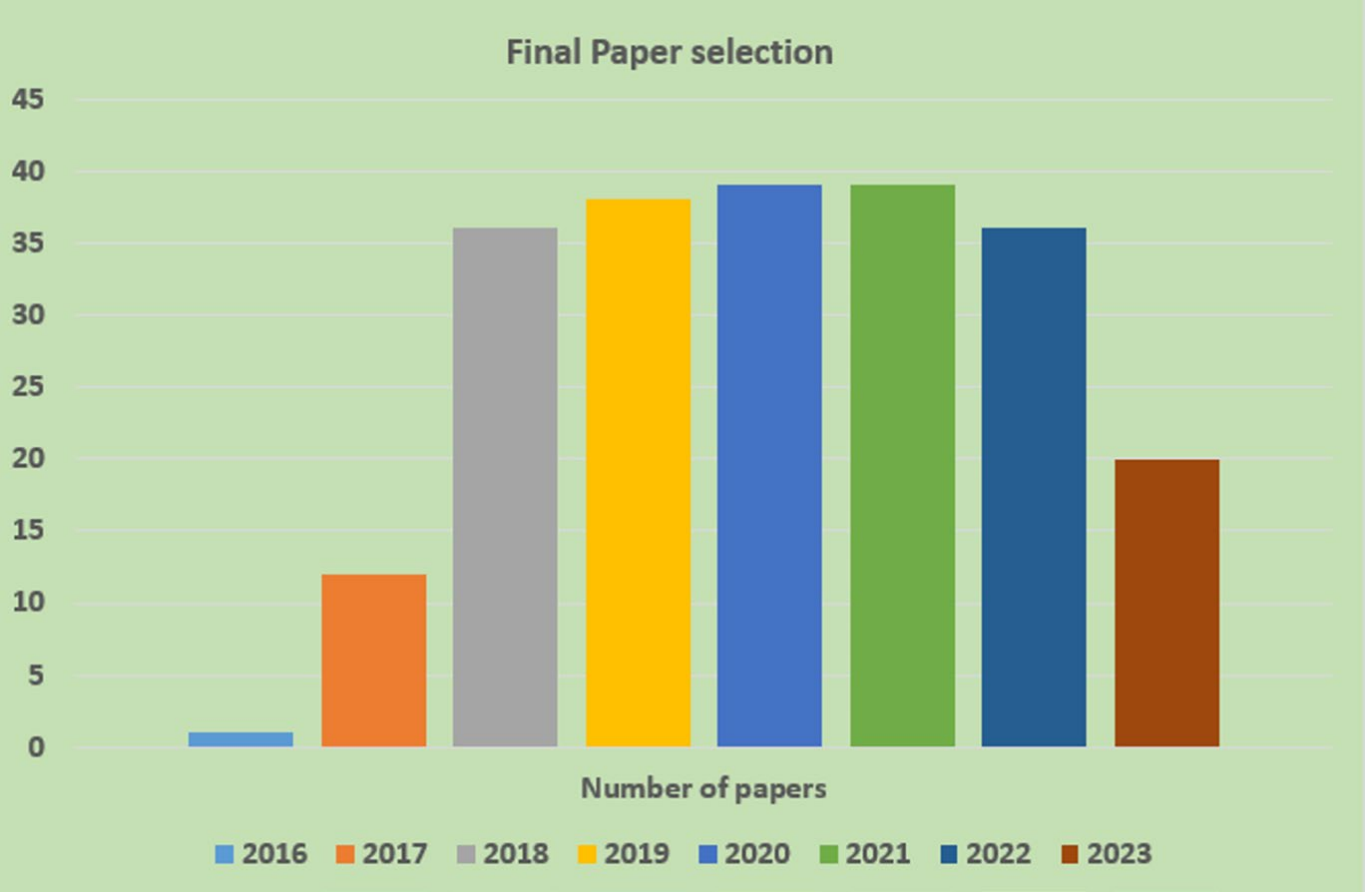
Final paper selection.

**Figure 7:**
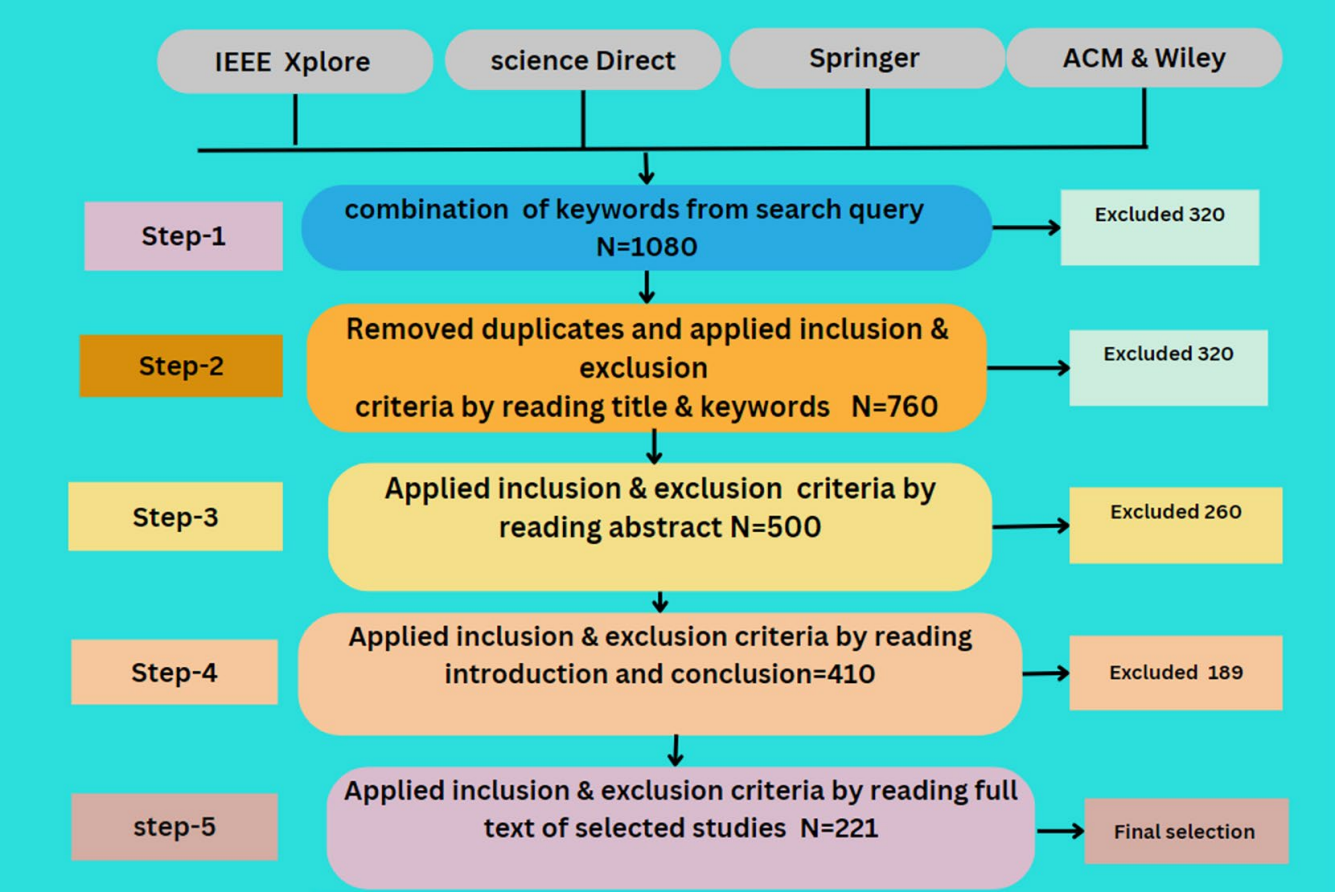
Prisma diagram.

Primary studies refer to the individual articles or book sections that directly address the research questions or topic of the review. This review has selected prior studies using the tollgate approach, a structured methodology of five phases^89^. This approach was instrumental in carefully curating 205 primary studies, considering the specified quality criteria for prior studies. The selection of papers is illustrated in Table 8 and Fig. 6. The prism diagram is shown in Fig. 7.

**Table 8:** Final paper selection.

Year	Number of papers
2016	1
2017	12
2018	36
2019	38
2020	39
2021	39
2022	36
2023	20

## Results

### Blockchain application, protocols, components, and types

A blockchain comprises a network of nodes that collectively maintain the ledger and collaborate to validate new transactions. Transactions are verified using sophisticated cryptographic techniques, ensuring their integrity and immutability. Blockchain technology has the potential to revolutionize various industries by enabling new forms of digital collaboration and innovation^90^. Blockchains can be either public, accessible to anyone, or private, restricted to authorized entities^91^.

**Figure 8:**
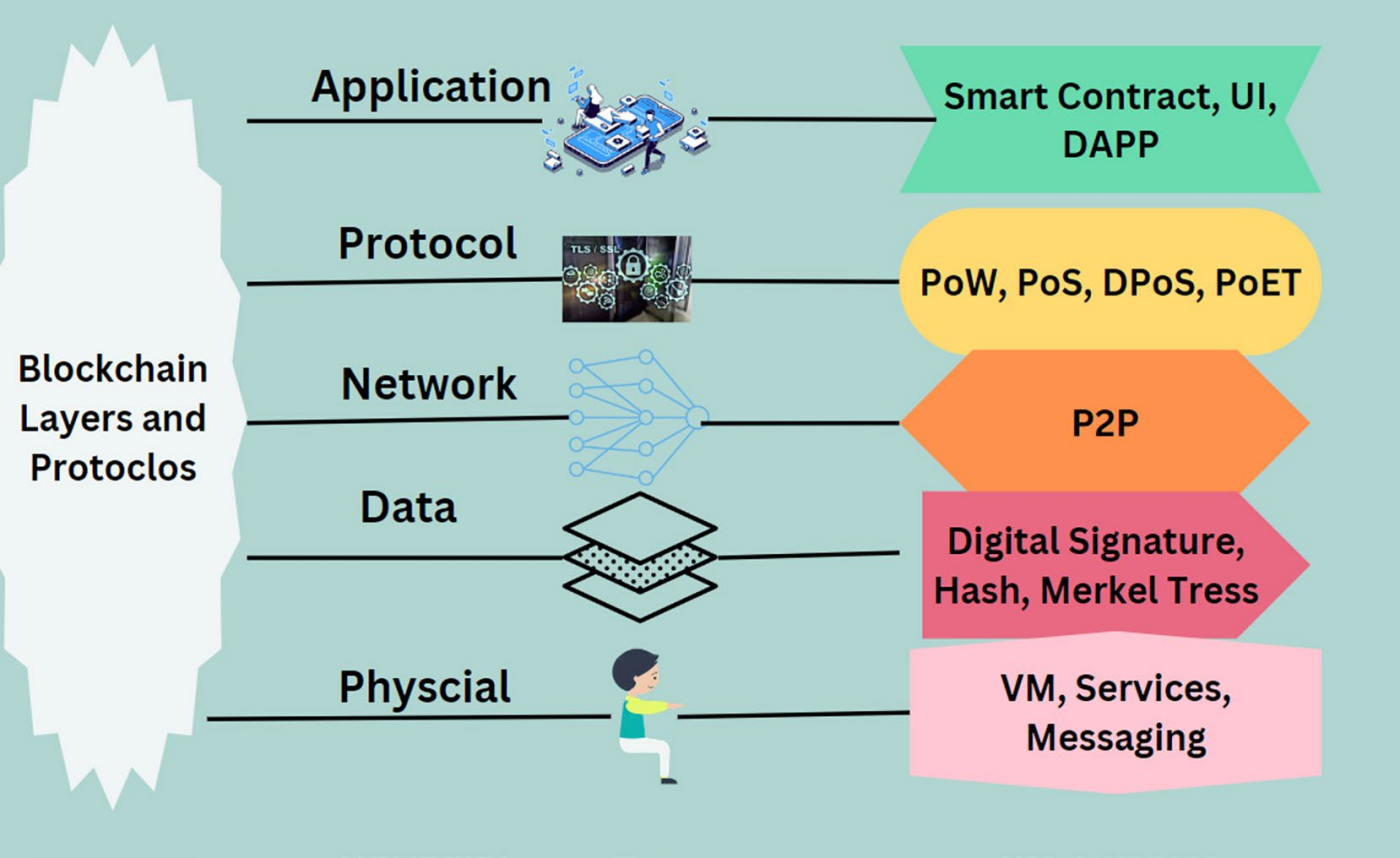
Blockchain layers and protocols^225^.

This section discusses the different layers of blockchain technology, each serving distinct functions within the system as illustrated in Fig. 2. These layers collectively provide the infrastructure, protocols, and applications that facilitate the blockchain's decentralized, secure, and transparent operations. Figure 8 offers an illustration to this aspect.

- Application Layer: This layer allows users and developers to engage with the blockchain for specific use cases, such as asset tracking, identity management, and financial services^92^. It includes applications and smart contracts that leverage the blockchain infrastructure. User interfaces, like wallets and browsers, enable interaction with the blockchain network in a user-friendly manner^93^.
- Protocol Layer: This layer defines the rules and procedures that govern the operation of the blockchain network^94^. It encompasses the blockchain's core software that implements consensus algorithms for transaction validation and smart contract execution^95^. The protocol layer also includes APIs and interfaces for developers to create decentralized applications^89^.
- Data Layer: Responsible for the storage and management of data on the blockchain, this layer uses data structures like Merkle trees and cryptographic techniques to secure transaction data^96^. It uniquely manages the consensus algorithms essential for validating transactions, distinguishing it from the protocol layer's role in overall network governance^97^.
- Network Layer: Manages node communication and connectivity within the blockchain network^98^. This layer utilizes protocols and algorithms that facilitate peer-to-peer interactions without intermediaries, employing standard protocols like TCP/IP and HTTP alongside blockchain-specific P2P protocols.
- Physical Layer: Comprises the actual infrastructure supporting the blockchain network, including hardware, data centers, and servers^99^. This layer provides the essential resources like computing power, storage capacity, and network bandwidth, and can be distributed across multiple nodes in a decentralized blockchain.

### Components of blockchain

Initially, because of their constrained computing power, bandwidth, and energy-saving requirements, IoT devices with limited resources were not recommended to use blockchain directly. Consequently, fog and cloud computing technologies have been developed to solve these problems and enable deploying blockchains in IoT systems^100^. Fog computing at the edge is being replaced increasingly by computing capacity as smartphone-related technologies develop. People no longer worry about running out of computer space or Internet connectivity. Through the use of a technique called a ledger, the blockchain records every interaction and event that has taken place. This software can store the specifics of every P2P transaction, including those in a database. There is lots of storage space, so this is possible.

**Figure 9:**
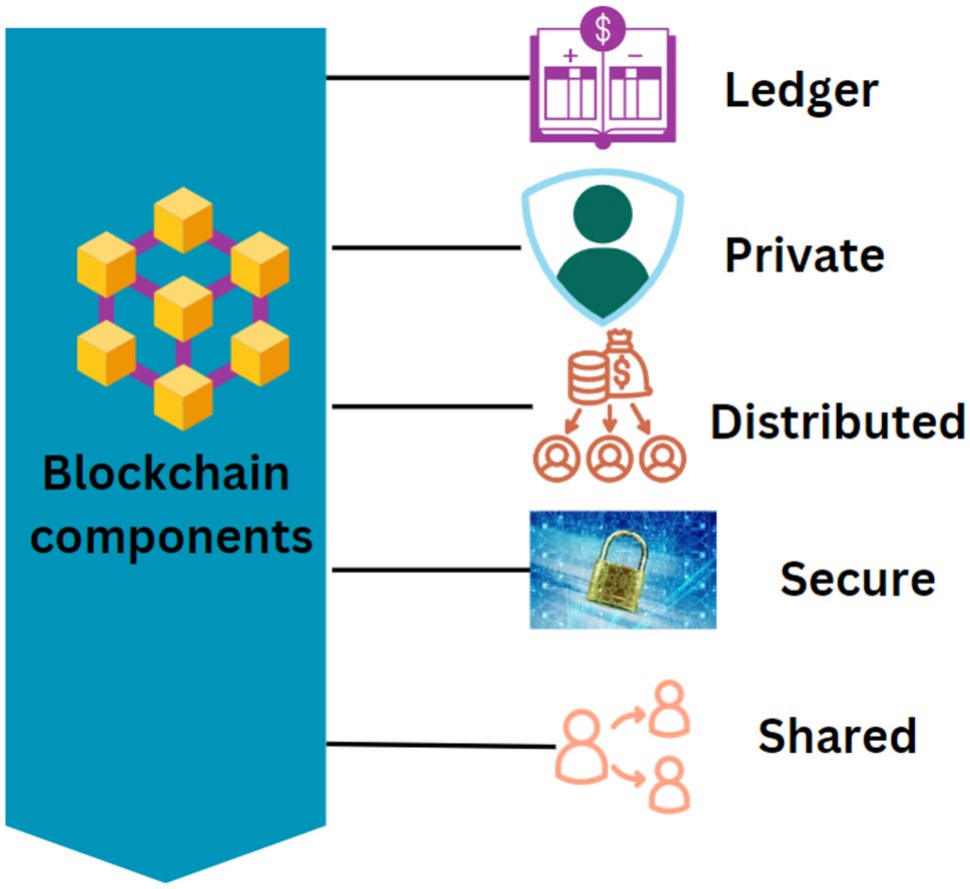
Overview of blockchain components: illustrates the key components of a blockchain, emphasizing its distributed architecture, scalability options, and security layers, pivotal for enhancing system robustness and security in applications like healthcare.

Moreover, it populates the database with data quickly and easily. The ledger follows the “no overriding” principle instead of conventional databases to guarantee that the blocks are almost always updated. By doing this, the system data is ensured accurate as it is transmitted to every blockchain node. As the network’s backbone technology, Hyper-ledger Fabric will be extensively used in our prototype. All blockchain events and related data are processed and stored through the decentralized hyperledger fabric architecture. Data can be sent from the cloud service provider’s analytics model to the hyperledger more easily when devices are recognized and a cloud gateway is used. The final agreement will then be able to be executed across the P2P network because of a smart contract implemented at the blockchain hyperledger level^101^. Because the system is distributed, it complies with federal privacy rules, including the Health Insurance Portability and Accountability Act of 1996 (HIPAA). Patients can rest easy knowing that their data is private and safe. Validation of the work Using cryptography, the blockchain network encrypts all the data it stores. These algorithms are discrete computer techniques controlled by hash functions and spatial mathematical processes. Information is stored and retrieved via these processes. Several security levels protect the ledger’s stored data from unauthorized access. These documents cannot be changed or examined by strangers. Any changes made to data that is available to the public must include the hash value. One major factor in a blockchain network’s security is its encryption feature. The ability of blockchain security to merge the hash values of the current block with those of the blocks preceding and succeeding it on the chain is an extra exciting feature^102^. We can confirm this hash value with every network node to know if it is accurate^103^. A disparity between the value shown and the actual value suggests that the data has been manipulated. The related data block has to be treated carefully. Put differently, the blockchain represents a secure approach to safeguarding user information. The section of the blockchain that stores P2P network data is called The Ledger. The network’s endpoints can hide their identities using secret hashing^104^. Since everyone has the same rights and skills, everyone can help ensure the security of the data. Figure 9 illustrates blockchain components. The distributed nature of the data across all global nodes makes it exceedingly difficult to refute or modify the unified model. Moreover, a distributed blockchain can be made. It is possible to change the relative sizes of the nodes that make up the blockchain^105^. As a result, the blockchain can improve its effectiveness, robustness, and resistance to crimes and cyberattacks. The blockchain’s consensus mechanism, one of its numerous security layers, might be harmed theoretically^106^. Private blockchains will be very helpful right now. A private blockchain will handle all communication between the health monitoring app and any other parties and between the patient and the applications.

### Types of blockchain

#### Public blockchain

Public blockchains operate as integral components of the public infrastructure, allowing individuals to use them without special permissions. All participants can observe and verify all transactions. Ethereum and Bitcoin are among the most extensively used public blockchain platforms. These networks epitomize transparency and openness, with network participants, commonly known as “miners,” incentivized to validate transactions^107^.

#### Private blockchain

Private blockchains are restricted to individuals who have received an invitation to join. These networks are designed for organizations that need to limit access to authorized personnel only, ensuring the confidentiality of data and transactions^108^.

#### Authorized blockchain

Authorized blockchains, similar to private networks, restrict user participation and the types of transactions that can be conducted. To participate, individuals must receive an invitation or obtain permission. These blockchains are typically managed by a single entity which oversees the permissions^35^.

#### Consortium blockchain

Consortium blockchains represent a collaborative approach, managed by a group of organizations rather than a single entity. These blockchains use reputation as a criterion for access and are particularly effective for transactions that require consensus among all participating entities. They offer a robust method for managing continuity in business transactions that involve shared responsibilities^35^. In such networks, consensus on transaction validity is reached collaboratively, ensuring that all entities involved can trust the recorded transactions^109,110^.

#### Hybrid blockchain

**Figure 10:**
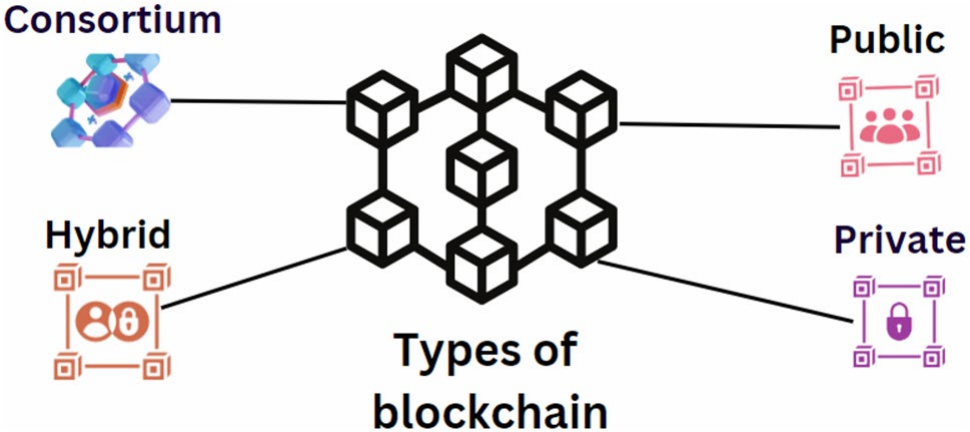
Types of blockchain: public, private, hybrid and consortium.

Hybrid blockchains combine elements of both private and public blockchains. They allow organizations to set up a private, permission-based system alongside a public, permissionless system that can interact with it. This setup ensures that organizations can control access to certain data while still enabling public verifiability where necessary. This section will reflect the detailed functioning of hybrid blockchains as illustrated in Fig. 10, which needs to be revised to accurately depict this description.

#### Practical byzantine fault tolerance (PBFT)

This ensures consensus even when a minority of individuals show unpredictable behaviour. A new block is appended when most verification pairs exceeding two-thirds result in the same conclusion^111^. The authors propose a reputation-based Delegated Byzantine Fault Tolerance (DBFT) consensus approach to determine the official blockchain efficiently. They provide a compelling illustration of this process. Several other consensus mechanisms are now being employed, including PoA^112^, Quorum Chain^8^, and Raft^113^. These phenomena are often viewed as a bit insignificant. A comprehensive examination of consensus approaches can be found in the publication^114^. Figure 10 illustrates the types of blockchains.

### Challenges in IoT-based medical systems

IoT devices face many security challenges, which can risk their users’ data and privacy. With different kinds of interactions that can take place in both the virtual and the real worlds, maintaining the safety of IoT systems is of extreme significance^115^.

#### Data privacy and scalability issues

IoT-based medical and healthcare systems handle compassionate data, such as patient medical records, biometric data, and personal identifying information. It is essential to ensure that this data is protected, encrypted, and not accessible by unauthorized parties^116^. IoT-based medical systems need to be able to manage a high number of data coming from various sources and devices,scalability is an essential issue that must be addressed^117^. Heterogeneity refers to the connectivity of IoT devices with distinct identities, various release versions, and distinct technological interfaces to carry out a wide range of tasks^118^. Because of this, the IoT has to deal with various devices and scenarios and link disparate networks and things^119^.

#### Network connectivity and autonomous management

IoT-based healthcare systems rely on network connectivity to transmit data from multiple devices to a central system. It can be challenging in areas with poor network coverage^120^. IoT involves making decisions and performing actions without human involvement. While technology can increase convenience and efficiency, it also raises serious security and ethical issues^121^. Devices in IoT systems are small and difficult to protect, such as fixed devices that are quickly damaged by natural disasters and can be stolen^122^.

#### Limitations in resources

IoT devices in healthcare can have resource limitations that can impact their performance and reliability^123^. IoT devices in healthcare often have limited memory, affecting their ability to store and retrieve data. It can be challenging when much data must be processed and analyzed^124^. Many IoT devices used in healthcare are battery-powered, which can impact their reliability and longevity. It is essential to ensure that these devices have adequate battery life to function reliably over extended periods^125^.

#### Security attacks on IoT systems

Security is a significant concern when it comes to IoT systems. Several security measures can be implemented to protect IoT systems from security to ensure the security of IoMT systems and protect sensitive patient data^126^. Data should be encrypted to prevent unauthorized access. Regular backups of all IoMT data should be maintained to ensure continuity of care in case of a security breach and system failure^127^. Here are some examples of security attacks in IoT systems.

#### DoS and Malware attacks

This attack sends many requests to the IoT device, making it unavailable or inaccessible. This can disrupt the device’s normal functioning or prevent it from communicating with other devices^128^. IoT devices may be vulnerable to malware that can infect the device and compromise its security. This can cause data loss, damage to the device, or even allow unauthorized access to the system^129^. In this attack, an attacker intercepts the communication between two IoT devices and can view, modify, or inject data into the communication. This can lead to unauthorized access or data theft^130^.

#### Data breaches

IoT devices store sensitive data such as user credentials, personal information, and other confidential data. If this data is not adequately protected, it can be compromised and stolen by attackers^131^. Data infusion is the process by which attackers transfer data while giving the impression that the data has a legitimate identity. When a DoS attack is launched against an IoT system, its resources are used to the point where they are unavailable to users^132^. IoT devices can also be physically tampered with, stolen, and damaged, leading to data breaches or loss of functionality^133^.

### IoT-based medical applications

#### Applications

**Figure 11:**
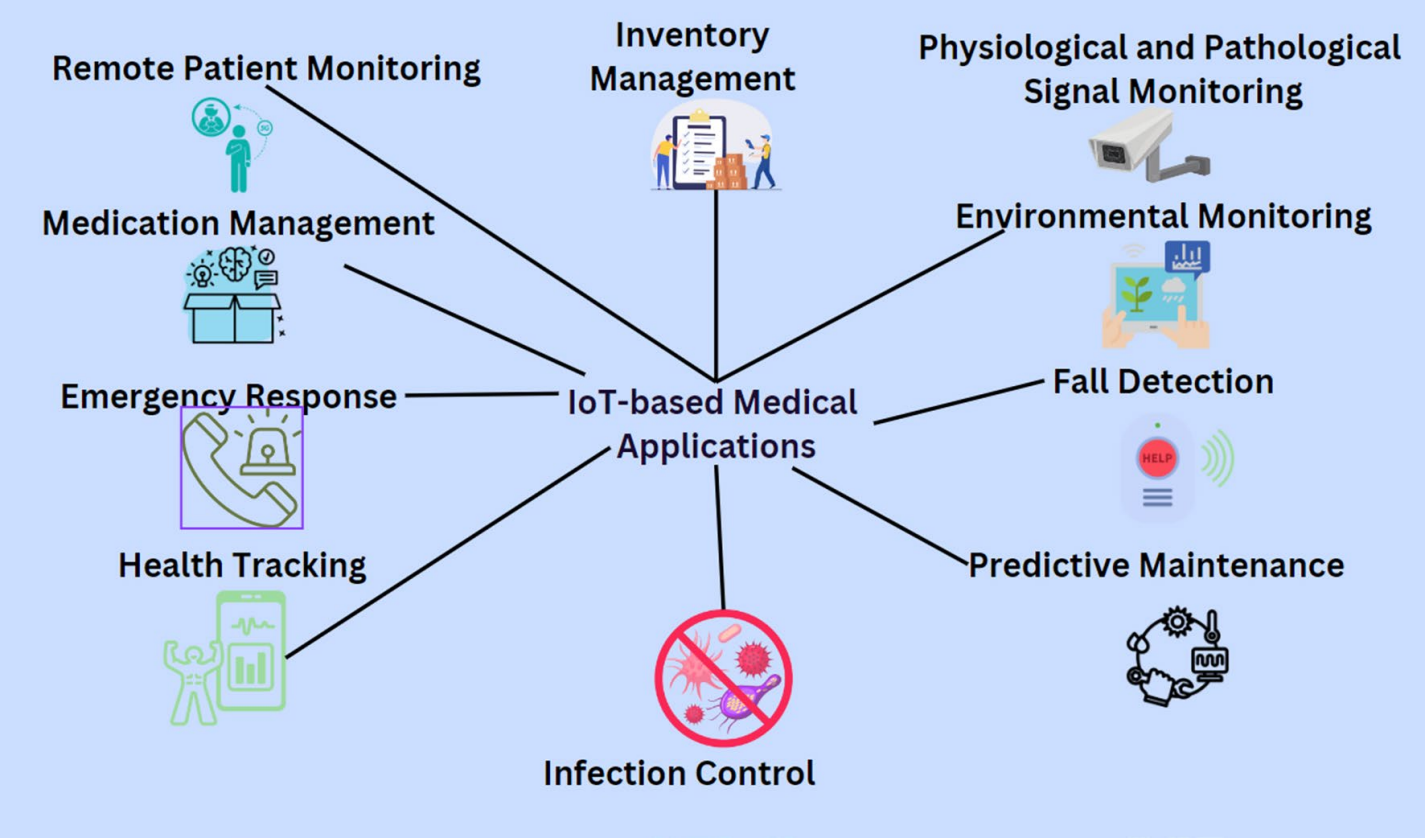
IoT-based medical applications.

Numerous IoT-based healthcare applications have the potential to transform the healthcare industry by enabling remote monitoring, reducing hospital readmissions, and improving patient outcomes, as mentioned in Fig. 11. IoT-based healthcare applications have the potential to revolutionize the healthcare industry by enabling remote monitoring, improving patient outcomes, and reducing healthcare costs^134^.

**A. Remote patient monitoring**

IoT devices such as wearables, sensors, and health monitors can remotely monitor patient health data such as blood pressure, heart rate, and blood sugar levels^135^. It enables doctors to provide timely interventions, reduce hospital readmissions, and improve patient outcomes.

**B. Medication management and rehabilitation**

IoT-based medication management systems can remind patients to take their medication and provide feedback to healthcare providers. It reduces medication errors and improves patient treatment plan adherence^136^. IoT devices can facilitate communication and coordination between healthcare providers, caregivers, and patients, allowing for a more collaborative and integrated approach to rehabilitation^137^. IoT devices can gamify rehabilitation by providing rewards and incentives for meeting goals and milestones, which can improve patient engagement and compliance^138^.

**C. Telehealth and health tracking**

IoT-based telehealth systems can enable remote consultations between doctors and patients, reducing the need for in-person visits. It can improve access to care, particularly for patients who live in rural or remote areas^55^. IoT devices can track health metrics such as sleep quality, activity levels, and calorie intake. It can provide patients with insights into their health and enable them to make informed decisions about their lifestyle^139^.

**D. Physiological and pathological signal monitoring**

Monitoring applications, such as creating numerical information and medical records linked to a medical condition, which can replace conventional hospital information systems, can be sponsored by a framework based on the IoT paradigm and combining technology for mobile communication^140^

**E. Hospital asset management and predictive maintenance**

IoT-based asset management systems can track the location and status of hospital equipment, such as infusion pumps and monitors. It can improve asset utilization and reduce the risk of misplaced equipment^141^. IoT-based predictive maintenance systems can monitor the health of hospital equipment and predict when maintenance is required. It can reduce downtime and improve equipment reliability^142^.

### Technologies that enable the protected IoMT

Global networks are the interconnected infrastructure for communicating and exchanging information across regions and countries^143^. Sensors in IoT devices are used to collect data from the physical world. Sensors are small devices that can detect and measure a particular physical quantity, such as temperature, humidity, light, motion, pressure, and sound^144^. IoT devices can incorporate one or more sensors and use them to collect data in real time.

#### Layers of IoMT

IoMT is a subset of the broader IoT ecosystem focused on medical and healthcare applications^144^. IoMT devices can include wearable devices, such as smartwatches and fitness trackers, and medical devices, such as glucose and blood pressure monitors. These devices can collect data on various health-related metrics, including heart rate, blood pressure, glucose levels, and physical activity^145^. IOMT has multiple layers, each of which plays a critical role in the overall system by understanding the different layers of the IOMT ecosystem^146^. Healthcare professionals and technology experts can work together to design and implement effective and secure IOMT solutions that improve patient outcomes, reduce healthcare costs, and enhance the overall quality of care^147^. These layers typically include the following.

**A. Sensing layer**

This layer includes the physical devices and sensors used to collect data from patients and medical devices. These sensors include wearable devices such as fitness trackers, smartwatches, and medical devices such as blood glucose monitors, electrocardiogram (EKG) machines, and blood pressure cuffs^147^.

**B. Network layer**

**Figure 12:**
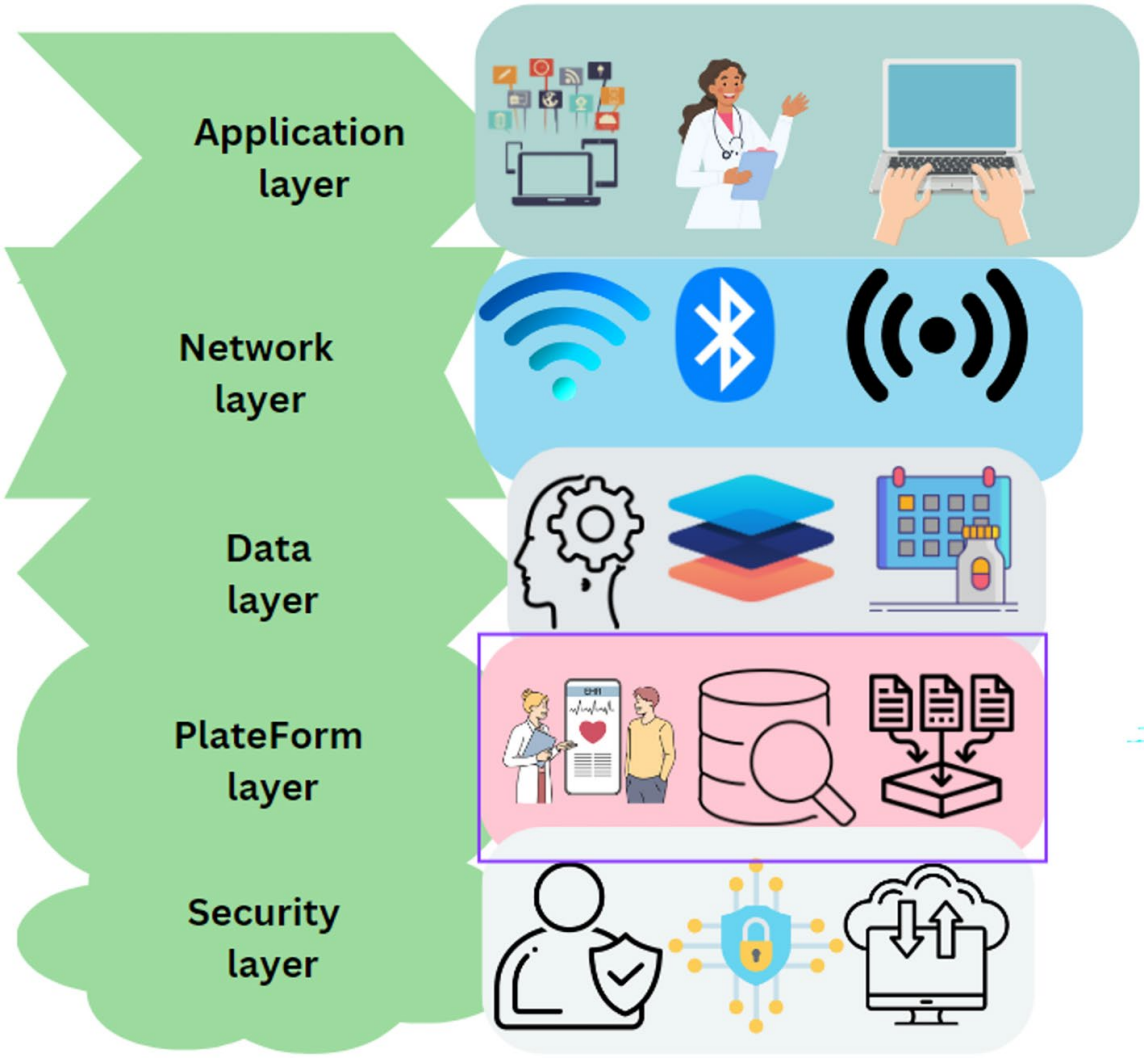
Layers details of IOMT and the input/output as well as the application related to each layer.

This layer comprises the communication infrastructure that enables data transmission from the sensing layer to other system parts. It can include wireless and wired networks and protocols such as Bluetooth and Wi-Fi^147^. Figure 12 illustrates layers of details of IOMT.

**C. Data layer**

This layer encompasses the data generated by IOMT devices. This data can include a range of health-related metrics, including vital signs, medication adherence, and activity levels^148^.

**D. Platform layer**

This layer comprises the software platforms and applications used to collect, store, and analyze IOMT data. This can include electronic health records (EHRs), health monitoring apps, and clinical decision support tools^149^.

**E. Security layer**

This layer ensures the confidentiality, integrity, and availability of IOMT data. It includes encryption, access controls, and data backup and recovery^126^.

### Integration of Blockchain with IoT applications to develop a secure IoMT

One of the key features of Blockchain technology is decentralization, which can help solve the single-point-of-failure problem in IoT systems. In a centralized system, all data and control are concentrated in a single point, such as a server on a data center^150^. This can create a single point of failure, where if the central point fails, the entire system may stop functioning. In a Blockchain-based IoT system, devices and sensors can communicate directly, and data can be stored on multiple nodes, ensuring redundancy and resilience. While data is loaded into the peer-to-peer blocks of a network, the Blockchain mechanism protects against fraudulent attacks and technological failure^151^. Blockchain technology provides an auditable log of events. This is achieved through the use of an immutable ledger that records all transactions in a transparent and tamper-proof manner^152^. Once a transaction is recorded on a Blockchain, it cannot be altered without the consensus of the network participants. This makes it possible to create a complete and auditable record of all events within the system, including transactions, ownership changes, and data updates^153^.

This feature has numerous applications in various industries, such as supply chain management, finance, and healthcare. In supply chain management, a Blockchain can be used to track the movement of goods and verify their authenticity. In finance, a Blockchain can create an auditable record of transactions and prevent fraud^154^. In healthcare, a Blockchain can securely store and share medical records, ensuring that patient data is accurate, up-to-date, and accessible only to authorized parties^155^. Blockchain technology uses immutable hash chains and digital signatures as critical components of its security and data integrity mechanisms. A hash chain is a series of hash functions applied to each block of data in the Blockchain. Each block in the chain contains a hash of the previous block, creating a chain of interlinked blocks^156^. This provides tamper-evident security because any change to a block in the chain will result in a different hash value, which the network will detect. Hash chains ensure the integrity and immutability of the data stored in the Blockchain^157^. Digital signatures are cryptographic techniques used to authenticate the origin of data.

**Figure 13:**
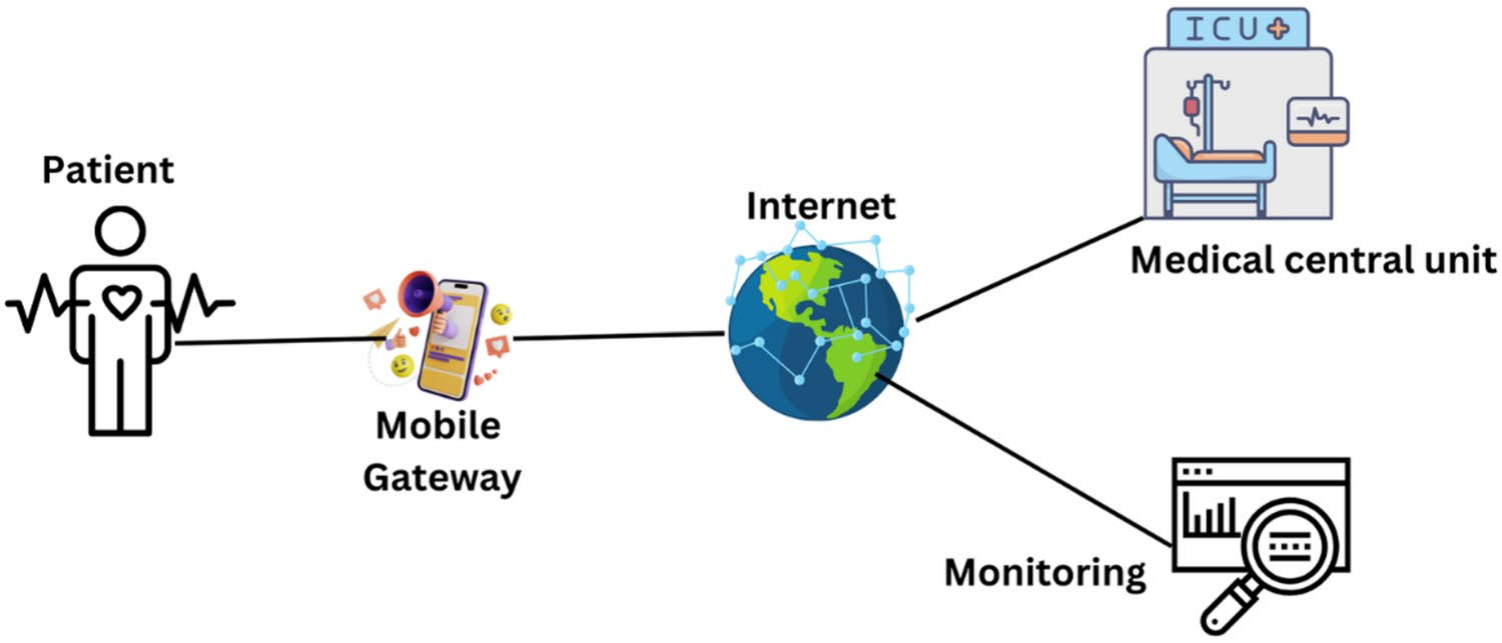
Blockchain and IoT convergence for secure IoMT: depicts how blockchain and IoT are integrated to enhance the security of the IoMT, highlighting the role of smart contracts in automating and securing healthcare processes.

Digital signatures are created using private and public keys, where the private key is known only to the signer and the public key is available to anyone. Only authorized parties can create and sign transactions on the Blockchain^158^. One of the key benefits of Blockchain technology in healthcare is its ability to ensure the privacy and security of patient data. Blockchain technology can provide secure and transparent access to patient records using a decentralized and encrypted ledger while ensuring that only authorized parties can access sensitive information^159^. This can help prevent data breaches and protect patients’ privacy. Another challenge in healthcare is interoperability. Blockchain technology can help overcome this challenge by providing a standardized platform for data sharing and communication across different healthcare systems^160^. This can improve the efficiency of healthcare delivery and enable better coordination of care. Fraud prevention is also a significant issue in healthcare. Blockchain technology helps prevent fraud by providing a secure and transparent way to track and verify transactions, ensuring that only legitimate claims are paid^161^. Gordon and Catalini^162^ used Blockchain to improve the patient-centric operational capability in medicine. Parallel healthcare system (PHR), which is based on artificial intelligence, uses computer experiments to connect hospitals, health bureaus, and patients so that data can be shared and medical records can be carefully looked over and evaluated^163^. Guard Time Health is a blockchain-based framework for healthcare data management and sharing. It is designed to provide secure and auditable access to patient data while maintaining the privacy and confidentiality of sensitive information^164^. Figure 13 shows the integration of blockchain with IoT to develop a secure IoMT. Smart contracts and blockchain technology can be used to maintain and manage PHI securely and transparently. Smart contracts are self-executing contracts programmed to automatically perform certain actions when conditions are met^164^. Smart contracts can automate various processes in healthcare, such as patient consent, insurance claims, and medical record sharing. By using smart contracts, healthcare providers can ensure that patient data is accessed and shared only with authorized parties and that patient consent is obtained and verified securely and transparently^165^. Smart contracts can also be used to enforce data access controls and data sharing agreements, helping to prevent unauthorized access to sensitive information.

### Blockchain in medical healthcare: use cases

#### Blockchain-based drug and medical management

Blockchain-based vital sign monitoring platforms provide a secure and transparent way to monitor and track patients’ vital signs in real-time while ensuring the privacy and confidentiality of patient data^166^. The platform consists of wearable devices and sensors that capture patients’ vital signs, such as heart rate, blood pressure, and respiratory rate. The data collected by these devices is transmitted to the blockchain network, where it is stored on an immutable and tamper-proof ledger. With blockchain technology, healthcare providers can ensure that patient data is secure and can only be accessed by authorized parties^167^. Cryptography techniques, such as public–private key pairs, can also help ensure the privacy and confidentiality of patient data^168^. The platform also uses smart contracts to automate processes such as data sharing and patient consent. A smart contract can be programmed to automatically share vital sign data with a healthcare provider or family member when certain conditions are met, such as a patient experiencing a sudden change in vital signs^169^. A framework that is based on cloud and is combined with Blockchain and IoT can provide a powerful platform for data sharing and management in various industries, including healthcare. In this framework, a cloud-based platform can store and manage data from multiple IoT devices, such as wearable health sensors or medical devices. The data can be transmitted securely to the cloud platform using encryption.

#### Blockchain-based mobile health

Blockchain technology is being used to enhance the security, privacy, and efficiency of mobile health (mHealth) platforms. Using blockchain, mHealth platforms provide a secure and transparent platform for managing patient health data and improving healthcare services^170^. With the decentralized blockchain network, patient data can be stored securely and accessed only by authorized parties. This can help prevent unauthorized access to patient data and ensure the integrity of the data^171^. Numerous researchers use blockchain to secure smartphone apps that collect data from patients’ wearable sensors and offer fast health services^172^. mHealth is based on a mobile app that uses JavaScript object notation (JSON) as its main programming language^173^. Blockchain is used in mHealth to protect health information by changing data obtained by wearable sensors^174^.

#### Blockchain-based access control security in E-health

Access control is an important security feature for e-health systems^175^. Access control refers to limiting and controlling who has access to sensitive health information stored within the system^12^. In an e-health system, access control may involve implementing various measures to ensure that only authorized individuals can view, modify, or delete patient health records^176^. This can include using secure login credentials, implementing role-based access controls, and tracking user activity to monitor for any unauthorized access attempts. A hypothetical e-health system built on blockchain technology and cloud effectively communicates health data to authorized users^177^. Blockchain’s technology solution can make current storage techniques, such as medical platforms that are traditional cloud IoT-enabled and electronic health records, safer and more effective for sensitive health data^82^. 10.4. Managing Medical Records and Other Data: there are several top blockchain-based frameworks for managing medical records and other data in healthcare. MedRec is a blockchain-based platform developed by researchers at MIT^178^.

The platform uses Ethereum blockchain technology to enable secure and transparent data exchange between patients, healthcare providers, and other stakeholders. MedRec enables patients to control their medical records and share them with healthcare providers as needed while ensuring the data is secure and tamper-proof^179^. Nebula Genomics is a blockchain-based platform that enables patients to store and share their genomic data securely. The platform uses blockchain technology^180^. Figure 5 shows different applications of blockchain.

#### Blockchain-based E-health smart contract

A healthcare framework based on Blockchain technology and smart contracts has the potential to provide a secure and efficient way to manage patient data. A framework known as HPA is a health prescriptions framework with special access for medical IoT devices^181^. These IoT devices are given an SAT, indicating that the IoT device has been authenticated and can make service requests to the network^182^. All patient data, including medical history, test results, and other relevant information, is stored on the blockchain. Each patient has a unique digital identity secured by a private key. A health chain is another blockchain-based platform that uses smart contracts to manage the sharing and access of patient data^183^. A hybrid health data platform was proposed by Alkhateeb et al.^184^ and combines a centralized data warehouse and a blockchain-based decentralized data repository. The centralized data warehouse stores data commonly used by healthcare providers, while the decentralized data repository stores more sensitive data, such as genomic data and personal health records^185^. The blockchain ensures the security and privacy of the decentralized data, and smart contracts are used to manage access and sharing permissions^186^. Blockchain-based electronic medical records frameworks can provide a secure and decentralized platform for storing and sharing patient health information^187^. Coral Health is a blockchain-based EMR framework designed to provide a secure and interoperable platform for healthcare providers to share patient data^188^. It uses a blockchain-based system to securely store patient data, with data encryption, access control, and tamper-proofing features.

#### Patient monitoring/electronic health record

EHRs are digital versions of a patient’s paper medical records. EHRs contain comprehensive and up-to-date information about a patient’s health, including medical history, diagnoses, treatments, medications, and test results^189^. This framework is a decentralized platform designed to enable secure and transparent data exchange between patients, healthcare providers, researchers, and other stakeholders. The platform offers secure and tamper-proof data storage, enabling fast data sharing between multiple parties^190^. Table 9 illustrates the role of blockchain in healthcare.

**Table 9:** Role of blockchain in health.

References	Applications	Summary
Ekblaw et al.^226^	Electronic health records	EHRs are securely stored on a distributed ledger utilizing a dependable blockchain infrastructure, ensuring their continuous safety and integrity throughout creation and access
Benchoufi et al.^227^	Clinical research	The utilization of blockchain technology establish a decentralized and secure frame-work for the exchange of clinical trial data. Technological advancements facilitate the ability to communicate data among research teams safely
Siyal et al.^228^	Medical fraud detection	The permanent nature of blockchain technology enhances fraud detection capabilities by preventing duplication or alteration of previously recorded transactions. Consequently, the implementation of open and truthful financial transactions is facilitated
Taherdoost^229^	Neuroscience research	Blockchain technology presents an exciting and innovative path for developing many applications related to brain training, modeling, and cognitive processes. The storage requirements for a complete replication of the human brain in digital form are significant, making blockchain technology an exciting and novel advancement
Plotnikov and Kuznetsova^230^	Pharmaceutical industry and research	Due to its inherent capacity for precise traceability, blockchain technology is very suitable for monitoring the pharmaceutical supply chain. The standard protocol involves verifying a drug’s origin, composition, and ownership to prevent theft

## Open challenges to the integration of Blockchain with the IoMT

**Figure 14:**
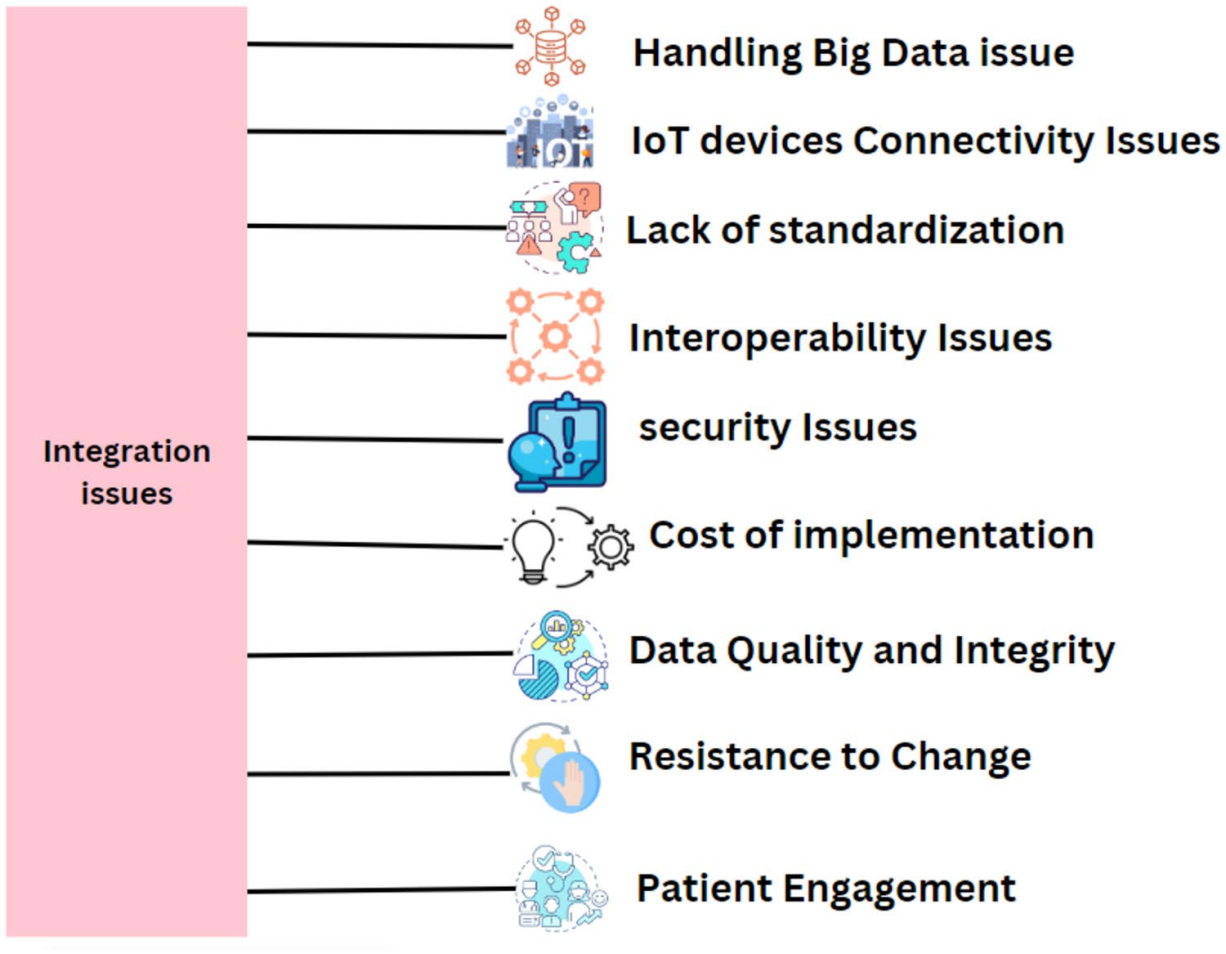
Integration issue^74^.

The challenges related to this section were covered by Megha et al.^191^. Let us synthesize the main results from this study, specifically addressing known challenges such as lack of standardization, scalability, interoperability, security, and skill levels in blockchain applications for healthcare:

- Lack of Standardization: The lack of standardized protocols and data formats across systems significantly hampers the integration of blockchain technologies in healthcare. Developing universal standards is critical for enabling seamless and effective implementations, which would foster broader adoption and interoperability across different healthcare systems and regions^192^.
- Scalability: The study discusses scalability challenges due to the vast and continuously growing volume of data in healthcare systems. It proposes solutions such as off-chain data handling and layer-two protocols to process transactions outside the main blockchain, which helps reduce load and improve transaction speeds (LaKeisha et al. 2014)^193^.
- Interoperability: Effective interoperability between different blockchain systems and existing healthcare information systems is essential for comprehensive healthcare delivery. The study reviews interoperable frameworks that allow systems to communicate and share information without compromising security or data integrity, enhancing collaborative healthcare efforts^194,195^. Different integration issues are illustrated in Fig. 14.
- Security: Security concerns are paramount due to the sensitive nature of personal health information. The paper reviews various cryptographic methods and consensus algorithms that enhance data integrity and confidentiality within blockchain networks. It also emphasizes the importance of robust access control mechanisms to prevent unauthorized data access while ensuring availability for authorized users^50,196^.
- Skill Levels: The adoption of blockchain technology requires significant skill upgrades among healthcare and IT professionals. The study advocates for targeted educational programs and workshops to improve understanding and operational capabilities regarding blockchain technologies within the healthcare sector^15,197^.

These areas highlight the critical challenges and potential solutions for integrating blockchain technology in healthcare. Addressing these issues is essential for leveraging blockchain to enhance data security, improve interoperability, and ensure scalability and standardization across healthcare systems.

## Solution of key challenges

### Managing scalability and storage requirements solution

Zero-knowledge proof (ZKP) is an encryption methodology employed to verify the authenticity of external entities. This cryptographic protocol ensures the long-term viability of the transmission while allowing the system to cover up the identities of the sender and recipient, as well as the content of the communication. ZKPs provide significant challenges in terms of programming, hence presenting a critical issue. The verification process between the sender and the recipient comprises several phases, with each successive round contributing to the cumulative number of rounds. The procedure faces additional expenditures due to the latency of the network and its limited potential for extension^198^. Accordingly, the capacity of large blockchain networks to process transactions continuously is limited because they can only validate a maximum of seven transactions per second. For lightweight nodes to effectively perform their designated tasks, others must possess all the required components and functionalities. A node is considered “full” when it encompasses all points inside its structure. While it is unnecessary for every lightweight node to store the entire blockchain, the growing quantity of these nodes may impose considerable strain on blockchain processors. The growth and data transmission processes for blockchain applications would encounter increased difficulty. Roehrs et al.^199^ proposed the utilization of Omni PHR as mentioned in their study. Patient health records (PHRs) can be organized in a manner that is both flexible and adaptable. A peer-to-peer network is employed to assist in the fragmentation of PHR into discrete data fragments. Furthermore, the design study shows that Omni PHR can ensure the uniform distribution of PHR data blocks across a routing overlay network. The concept for Health Chain was formulated by Ahram and his colleagues in the year 22. The emergence of blockchain technology has been identified as the underlying factor in this phenomenon. Notably, the modular architecture of 986 Hyperledger fabric stands out as its most prominent attribute, enabling enhanced security, scalability, and privacy within health informatics. Smart contracts are employed to define the associated rights and ensure that only authorized individuals are granted access to the network. The caching system developed by ???? was built around FPGA technology, primarily chosen for its advantageous characteristics of low power consumption and minimal resource demands. During periods of increased workload, the central processing unit (CPU) exhibited a raised energy consumption and resource utilization to store data effectively. The utilization of Field-Programmable Gate Arrays (FPGAs) in SHA-256’s implementation to address the issue of scalability and its capacity to reduce server load even under conditions of significant job volume is remarkable. This methodology effectively reduces the dimensions and energy consumption of the FPGA while enhancing its overall performance. The results yielded a significant decrease of 10 times in the required workload.

### Lack of standardization solution

Everyone must know and follow the rules because the approach might not work without clear standards and guidelines. HIPAA requirements must also be followed to guarantee that the correct data is utilized^200^, particularly in light of the regulations’ complexity and the ongoing development of new laws, standards, implementation guidelines, and cross-policy initiatives. Two countries and organizations that have supported and provided funding for state-run blockchain-based healthcare systems are Estonia and the United States. Improvements in blockchain, AI, and cloud computing will open up an infinite number of new opportunities, especially in the medical industry. The investigation results recommend several essential actions, some mentioned below.

- Analyzing the blockchain-based healthcare system’s most important component
- A local and worldwide risk study of the application of blockchain technology will be used to assess the security and resilience against new platforms, intellectual property insecurity, regulatory ambiguity, and strategic issues of the proposed blockchain-based system^201^.
- The third step of a blockchain cost–benefit analysis is to identify the features that set apart each potential and current design alternative and start building a model for each one according to its special qualities^202^.

### Interoperability solution

Developing comprehensive interoperability within the healthcare industry poses significant challenges because of the inherent vulnerability of patient privacy. Blockchain technology is rapidly developing as a beautiful solution for preserving functionality in the healthcare system. The highest priority is establishing strategies to enhance communication among care-related information systems^203^. It is imperative to ensure an adequate level of security during the process of transmitting patient medical records. Various risks linked to the practice give rise to potential consequences that can significantly affect healthcare providers’ financial, insurance, and ethical aspects^204^. Healthcare professionals often examine healthcare from various perspectives daily. One of the options available is utilizing Blockchain technology to operate a Hospital Management System (HMS). The objective is to enhance the safeguarding of information and simplify inter-system communication. In a healthcare environment, protecting user privacy, availability, and integrity is imperative while accessing information consistent with many systems. Achieving an equitable relationship between interoperability and security within the healthcare domain necessitates a careful balance due to the crucial functional basics, non-functional criteria, and business factors involved. Integrating BC technology in healthcare presents advantages and disadvantages, primarily due to a lack of skilled experts in developing effective software designs that fit this unique environment^205^. It is imperative to consider technological and conceptual solutions to effectively include trade-offs such as security and interoperability in the design process. The assurance of safety and interoperability in blockchain technology can be achieved by utilizing the Model-Driven Engineering (MDE)^206^ Framework. The underlying structure of this system is rooted in a complex architecture, with its primary objective being the provision of a distinct domain-specific language (DSL)^207^ for the specification of SC, which is separate from BC. This phenomenon enhances the level of interoperability and security within the healthcare system. To improve comprehension of the design and ensure the inclusion of all components, it is recommended to conduct an experiment employing the MDE. Interoperability holds significant importance within the healthcare industry, particularly in the exchange of EHRs. Obtaining patient information has become efficient and straightforward, reducing the possibility of errors.

- One potential area of focus for improvement is enhancing the productivity of the healthcare staff.
- A decrease in healthcare costs.
- Transactions that possess the characteristic of recovery and are impervious to alteration

### Security issues solution

The implementation of blockchain technology has the potential to address interoperability challenges within the healthcare system, thereby ensuring that patients remain the focal point of care^208^. This system preserves patient privacy, enhances communication efficiency within the healthcare system, and focuses treatment efforts on the individual patient^209^. The irreversible nature of blockchain technology has the potential to improve the precision of diagnoses, particularly in environments where protecting user privacy and safety is of utmost (importance^50^). Due to the prevalence of security issues related to healthcare data, individuals are increasingly expressing concern regarding the preservation of patient privacy and data integrity. Beazley, a globally recognized insurance enterprise with expertise in cybersecurity, reported that in 2017, approximately 45 percent of ransomware attacks were directed toward healthcare organizations^210^. The incidence of medical security breaches and theft of medical records is increasing. According to a report published by the HIPPA Journal^211^, the healthcare sector saw over 350 breaches in 2017. The number of violations in 2009 was less than twenty, indicating an upward trend. The recorded maximum value of the day before was below twenty. Utilizing blockchain technology has promise in enhancing identity management and data protection within the healthcare industry^209^. There are fewer potential risks, and unauthorized entities cannot gain access to private information. The process of encrypting data contributed to a blockchain involves the utilization of cryptographic techniques. This process renders the stored data irreversible and unavailable. To facilitate transaction processing, each user is assigned a private key, which serves as a means of authentication before transaction execution. Without explicit authorization, healthcare professionals such as physicians or nurses cannot retrieve a patient’s medical information stored on the blockchain^212^. Enhanced data sharing between healthcare providers increases the probability of achieving an accurate diagnosis and enhances the effectiveness of therapy. This enables healthcare facilities to provide cost-effective and efficient medical services. Utilizing blockchain technology allows patients to maintain privacy while sharing their data with any chosen service provider^213^.

## Comparison of existing blockchain technologies

### Blockchain implementations

Pflanzner et al.^214^ conducted a research study to compare the execution times, latency, throughput, and scalability of two different versions of HLF (v0.6 and v1.0). They tested with different workloads and node scales to test HLF v1.0, and they found that it consistently outperformed HLF v0.6 in every performance measure they looked at. Pflanzner et al.^214^ also looked at the throughput and latency of HLF v1.0 using an experimental methodology. They configured several transaction and chain code parameters using Caliper, the benchmarking tool, to examine these factors’ impact on transaction latency and throughput when dealing with micro-workloads. The results showed that the order’s parameters significantly affected HLF v1.0’s throughput. It cannot process transactions in parallel, which limits its ability to utilize many virtual CPUs and was found to be a severe drawback. Nguyen et al.^215^ experimented to investigate the effects of considerable network delays on fabric performance. HLF v1.2.1 was implemented in the experiment over a France-Germany area network. The results showed that considerable network latency negatively affected consistency guarantees, making HLF version 1.2.1 unsuitable for critical scenarios like trading or banking. Wang and Chu^216^ looked into Ethereum performance research on a private blockchain, focusing on the Pow-based Geth and PoA-based Parity clients. After their investigation, they found that Parity exceeded Geth in speed, processing transactions 89.82% faster under a range of workload variations. Nguyen et al.^215^ experimented to investigate the effects of large network delays on fabric performance. HLF v1.2.1 was implemented in the experiment over a France-Germany area network. The results showed that considerable network latency negatively affected consistency guarantees, making HLF version 1.2.1 unsuitable for critical scenarios like trading. Wang and Chu^216^ looked into Ethereum performance research on a private blockchain, focusing on the PoW-based Geth and PoA-based Parity clients. After their investigation, they found that Parity exceeded Geth in speed, processing transactions 89.82% faster under a range of workload variations. Yasaweera singhelage et al.^217^ reported a technique that predicts the latency of systems based on blockchain using simulation and the Palladio Workbench. They measured latency on a private Ethereum (Geth) experimental environment by employing this method, and they were able to achieve a low relative error in response time typically less than 10%. Fan et al.^218^ conducted a comparative analysis of three distinct cryptocurrencies after Blockbench was developed: Ethereum (geth v1.4.18), Parity (v1.6.0), and HLF (v0.6.0-preview). They found that HLF is superior to Ethereum and Parity in macro and micro benchmarks. However, they found out that HLF had issues scaling up to 16 nodes. In addition, it was found that consensus procedures were the hurdles for Ethereum and HLF. Using a specially created workload, the evaluation of HLF v0.6 with PBFT, HLF v1.0 with BFT-SMaRt, and Ripple was conducted^219^. This was done to help with the challenge of comparing many blockchains. The results showed that although these blockchains have limited scalability, they offer a respectable throughput. Pongnumkul et al.^220^ conducted an initial performance assessment of HLF (v0.6) and Ethereum (geth 1.5.8) under varying workloads, and the results showed that HLF performs better than Ethereum. Both platforms, however, were shown to be superior compared to conventional database systems, especially when subjected to high workloads. Pandey et al.^221^ created the open-source simulation tool BlockSIM for private blockchain systems to help architects understand and plan the operational performance of such systems. Comparisons with a real-world private Ethereum network were used to show off BlockSIM’s efficiency. Alharby and van Moorsel^222^ created BlockSim, a configurable discrete-event simulator to analyze various blockchain implementations. They came to a few interesting findings from Ethereum and Bitcoin simulations. Similarly, DAGsim, a framework for continuous-time, multi-agent simulation applications for distributed ledgers based on DAGs, was introduced by Zander et al.^223^. This method provides information about IOTA’s performance. Analytical results were combined with experimental simulations in other studies. The authors used Python to build a DAG-based cryptocurrency simulator to verify an analytical performance model. This simulator provided information about transaction processing speed. Rochman et al.^224^ examined the effects of different tip selection strategies on the growth of the IOTA tangle using continuous-time simulations and a range of transaction arrival rates.

### Performance measurements

Some evaluations are explicitly dedicated to assessing the fine performance of individual steps, such as the efficiency of encryption and hash functions. These evaluations are in addition to complete end-to-end performance measurements. Equation (1) determines the time it takes to process a transaction.

1 $$T={t}_{i}+ {t}_{c}={(t}_{v}+ {t}_{pow}+{t}_{n}+ {t}_{e})+ {t}_{c}$$

The speed at which distributed ledger technology (DLT) transactions are mainly generated depends on the efficiency of the hashing and encryption algorithms. The following terms are included in the equation that is provided: t_i_ denotes the issuance time, t_c_ the confirmation time, t_v_ the validation time, t pow the proof-of-work time, t_n_ the network overhead, and the processing overheads, which include hashing, encryption/decryption, and authentication. The speed at which distributed ledger technology (DLT) transactions are mainly generated depends on the efficiency of the hashing and encryption algorithms. Blockchain technology uses two encryption algorithms: elliptic-curve cryptography (ECC) and rival Shamir Adleman (RSA). Their thorough analysis, which considered key size, key generation performance, and signature verification performance, showed that the ECC algorithm used by Bitcoin and Ethereum performs better than RSA in most cases. The study shows that ECC is more effective than RSA when it comes to meeting the security needs of blockchain technology. The authors explicitly created a blockchain in an IoT scenario to evaluate various cryptographic hash functions. The hash algorithms MD5, SHA-1, SHA-224, SHA-384, and SHA-512 are among them. The test findings show that SHA-224 and SHA-384 are the hash algorithms most suited for blockchain since they resist collision attacks. Collision attacks, which happen when two different messages generate the same hash, can break hash cyphers. This raises several red flags. Furthermore, it has been demonstrated that these two hash functions execute blockchain operations faster than other hash functions in terms of time.

## Conclusion

In conclusion, this study has comprehensively explored integrating blockchain technology with the IoMT in the healthcare sector. The study has made significant contributions in several key areas through meticulous research and analysis. The study has proposed using zero-knowledge proofs (ZKPs) to address the challenges of scalability and storage requirements. The study offers a viable solution to manage the ever-expanding data generated by IoMT devices by employing encryption techniques and efficient data fragmentation. It highlights the need for adherence to regulations like HIPAA and the role of blockchain in ensuring compliance. It explores the role of blockchain in enhancing communication among healthcare information systems while maintaining patient privacy. This study offers valuable insights into the potential of blockchain technology to revolutionize the healthcare industry by addressing critical challenges and providing secure, interoperable, and compliant solutions. The findings underscore the need for further research and implementation of blockchain-based systems in healthcare to improve patient care, reduce costs, and enhance data security. As the healthcare landscape evolves, blockchain technology is poised to play a pivotal role in shaping its future.

## Data Availability

The datasets used and/or analysed during the current study available from the corresponding author on reasonable request.
