# Author Correction: The role of blockchain to secure internet of medical things

**DOI:** 10.1038/s41598-024-71990-3

**Published:** 2024-09-12

**Authors:** Yazeed Yasin Ghadi, Tehseen Mazhar, Tariq Shahzad, Muhammad Amir khan, Alaa Abd‑Alrazaq, Arfan Ahmed, Habib Hamam

**Affiliations:** 1 Department of Computer Science and Software Engineering, Al Ain University, Abu Dhabi, 15322 UAE; 2 Department of Computer Science, Virtual University of Pakistan, Lahore, 55150 Pakistan; 3 Department of Computer Science, COMSATS University Islamabad, Sahiwal Campus, Sahiwal, 57000 Pakistan; 4 School of Computing Sciences, College of Computing, Informatics and Mathematics, Universiti Teknologi MARA, 40450 Shah Alam, Selangor Malaysia; 5 AI Center for Precision Health, Weill Cornell Medicine-Qatar, Doha, Qatar; 6 Faculty of Engineering, Université de Moncton, Moncton, NB E1A3E9 Canada; 7 School of Electrical Engineering, Department of Electrical and Electronic Engineering Science, University of Johannesburg, Johannesburg, 2006 South Africa; 8 Hodmas University College, Taleh Area, Mogadishu, Somalia; 9 Bridges for Academic Excellence, Tunis, Tunisia

Correction to: *Scientific Reports* 10.1038/s41598-024-68529-x, published online 08 August 2024

The Funding section in the original version of this Article was incomplete.

Funding

“This research work is supported by the Qatar National Library.”

now reads

“The publication of this article was funded by the Weill Cornell Medicine—Qatar Health Sciences Library.”

The original Article has been corrected.

